# The SLS-Berlin: Validation of a German Computer-Based Screening Test to Measure Reading Proficiency in Early and Late Adulthood

**DOI:** 10.3389/fpsyg.2019.01682

**Published:** 2019-08-14

**Authors:** Jana Lüdtke, Eva Froehlich, Arthur M. Jacobs, Florian Hutzler

**Affiliations:** ^1^Department of Experimental and Neurocognitive Psychology, Freie Universität Berlin, Berlin, Germany; ^2^Center for Cognitive Neuroscience Berlin, Freie Universität Berlin, Berlin, Germany; ^3^Centre for Cognitive Neuroscience, Department of Psychology, Paris Lodron University of Salzburg, Salzburg, Austria

**Keywords:** reading proficiency, screening test, aging, life span, sentence reading, eyetracking, text comprehension

## Abstract

Reading proficiency, i.e., successfully integrating early word-based information and utilizing this information in later processes of sentence and text comprehension, and its assessment is subject to extensive research. However, screening tests for German adults across the life span are basically non-existent. Therefore, the present article introduces a standardized computerized sentence-based screening measure for German adult readers to assess reading proficiency including norm data from 2,148 participants covering an age range from 16 to 88 years. The test was developed in accordance with the children’s version of the *Salzburger LeseScreening* (SLS, Wimmer and Mayringer, 2014). The SLS-Berlin has a high reliability and can easily be implemented in any research setting using German language. We present a detailed description of the test and report the distribution of SLS-Berlin scores for the norm sample as well as for two subsamples of younger (below 60 years) and older adults (60 and older). For all three samples, we conducted regression analyses to investigate the relationship between sentence characteristics and SLS-Berlin scores. In a second validation study, SLS-Berlin scores were compared with two (pseudo)word reading tests, a test measuring attention and processing speed and eye-movements recorded during expository text reading. Our results confirm the SLS-Berlin’s sensitivity to capture early word decoding and later text related comprehension processes. The test distinguished very well between skilled and less skilled readers and also within less skilled readers and is therefore a powerful and efficient screening test for German adults to assess interindividual levels of reading proficiency.

## Introduction

Reading and comprehending written words and texts is an essential skill for an active and self-determined participation in everyday life. Successful reading includes basic decoding skills, often investigated by means of single word recognition (e.g., Jacobs and Grainger, 1994; Ziegler et al., 2001). It also relies on higher cognitive processes such as integration of syntax and general comprehension, which is mainly studied using longer passages or texts (e.g., Graesser et al., 1994; Singer et al., 1994; Myers and O’Brien, 1998). Reading also includes affective and aesthetic processes that have been neglected by mainstream reading research but now get more into focus in the emerging perspective of neurocognitive poetics (Lüdtke et al., 2014; Jacobs, 2015a, b, 2017; Lüdtke and Jacobs, 2015; Willems and Jacobs, 2016; Jacobs and Willems, 2018). Not surprisingly, the most common standardized screening procedures applied in reading research and educational settings focus mainly at the first two levels, i.e., reading abilities of participants are assessed via tests, which are based on recognizing words/naming letter strings [e.g., SLRT-II (Moll and Landerl, 2010), TOWRE (Torgesen et al., 2012)] or on answering comprehension questions related to text passages [e.g., Nelson-Denny Reading Test (Brown et al., 1993), ELFE-II (Lenhard et al., 2018)]. Only very few tests use single sentences to evaluate reading skills, such as the Sentence Reading Fluency test from the Woodcock-Johnson IV: Tests of Cognitive Abilities (Schrank et al., 2014) or the SLS 2-9 (Wimmer and Mayringer, 2014). Given that words, on the one hand, and texts, on the other hand represent a continuum of potential reading material, single sentences represent an intermediate form of written input. Basic decoding skills as well as aspects of integration and comprehension converge at this intermediate level. Thus, single sentences are well-suited for measuring reading proficiency including reading fluency, i.e., the ability to read accurately and at a rate that enables comprehension (Cunningham, 2001; Wolf and Katzir-Cohen, 2001). The importance for investigating reading abilities with words, sentences and texts is reflected in the number of well-established and broadly used tests that exist for English-speaking research participants. These existing tests cover all three levels and offer norm data for the whole life span (e.g., Woodcock-Johnson IV: Tests of Cognitive Abilities, Nelson-Denny Reading Test). However, when it comes to German-speaking participants, there is an apparent lack of comparable screening measures, especially when considering younger and older adults. The absence of such tests, particularly for German (older) adult readers, is even more astonishing, given the importance of skilled reading for its (potential) role for the development and preservation of verbal memory processes (Demoulin and Kolinsky, 2016) as well as for maintaining functional independence (in old age; e.g., Meyer and Pollard, 2006). Moreover, failing to provide adequate screening tests addressed to a German-speaking population may potentially endanger research within that area. Therefore, the aim of the present paper is to introduce a standardized computerized sentence-based screening measure for German adult readers (ages: 16–88 years), which can easily be implemented in any research setting in German orthography and which provides norms for reading proficiency based on 2,148 participants.

### Reading Words, Sentences, and Texts

Reading proficiency is a complex, highly automatized multicomponent skill which combines a person’s ability to successfully process early word-based information and utilize this information in later processes of sentence and text comprehension. Early reading processes involve the effective interplay of recognizing single words and efficient eye movement control (Jacobs and Ziegler, 2015). More precisely, they include sublexical and lexical processing of orthographic, phonological and semantic information (Ziegler et al., 2008; Froehlich et al., 2016). Later processes related to the comprehension of sentences and texts contain component processes like deeper lexical, syntactic, and thematic analysis (Carpenter et al., 1995). All of them are necessary to construct representations of the information described in the reading material and enable understanding, enjoying and remembering (Rayner and Reichle, 2010; Jacobs, 2015a). According to the construction-integration model (Kintsch, 1988, 1998), proficient readers form three types of text representations, the surface form, the textbase and the situation model. While the surface level addresses word meaning and the syntactic form of the text, the textbase refers to the semantics of the text in form of propositions, that describe explicitly stated interrelationships of the given facts or ideas. The construction of a situation model requires a reader to integrate prior knowledge with the new information to build a coherent representation of the situation outlined by the text (e.g., Zwaan and Radvansky, 1998). Research has shown that situation models are created when reading single sentences (e.g., Radvansky and Dijkstra, 2007), i.e., already at the sentence level readers make use of preexisting pragmatic representations to facilitate comprehension (Carpenter et al., 1995). Processing sentences requires the reader to not only decode and assign meaning to words but also to make use of syntax, grammar, context (McClelland et al., 1989) and affective semantic information (Lüdtke and Jacobs, 2015). Furthermore, extra time is additionally allocated for conceptual integration at the end of sentences (Stine-Morrow et al., 2008). Besides, reading depends on accurate eye movement control and draws on a number of other fundamental cognitive and affective functions, such as attention, processing speed and working memory (Radvansky and Copeland, 2001; Reichle et al., 2003; Stine-Morrow et al., 2008), or mental simulation, empathy, immersion, affective evaluation and aesthetic appreciation (Willems and Jacobs, 2016; Jacobs and Willems, 2018).

To grasp and explain a proficient reading process, a number of computational models have been put forward at word (e.g., Grainger and Jacobs, 1996; Jacobs et al., 1998; Hofmann and Jacobs, 2014), sentence (McClelland et al., 1989), and text levels (e.g., Kintsch, 1988; Tzeng et al., 2005). Crucially, word identification models focus on early, basic processes of reading, whereas text comprehension models center largely on later processes establishing local and global coherence (Rayner and Reichle, 2010). The interaction of those early and late processes marks successful reading. Thus, the intermediate character of sentences provides an ideal way to assess reading proficiency, i.e., the quality with which the integration of early and late processes takes place.

### Assessing Individual Differences in Reading Across the Life Span

Besides being easily implemented and evaluated, using sentences to assess reading proficiency has the advantage that early basic reading processes are not necessarily masked by later comprehension processes. Not surprisingly, tests based on sentence reading have been developed both in English (e.g., Sentence Reading Fluency Test from Woodcock-Johnson IV) and German to assess reading development in children. One widely used screening test for German speaking children is the Salzburger Lese-Screening (SLS; SLS 1-4: Mayringer and Wimmer, 2003; SLS 5-8: Auer et al., 2005; SLS 2-9: Wimmer and Mayringer, 2014). The SLS measures basic reading ability in a natural environment with special emphasis on reading speed by simultaneously assessing reading comprehension. In this paper and pencil test, children are asked to silently read simple sentences and to mark them as true or false depending on the plausibility of the sentence (e.g., “Tomatoes are blue.” from SLS 2-9, Wimmer and Mayringer, 2014). The screening consists of 100 sentences (SLS 2-9; 70 for the SLS 1-4 and 5-8) and is administered within a 3 min time span. The SLS has a good parallel test reliability (SLS 2-9: *r* = 0.95 and *r* = 0.87 for second and eighth graders, respectively). SLS scores correlate well with the speed of reading aloud (SLS 2-9: *r* = 0.80–0.89 and *r* = 0.49–0.55 for second and eighth graders, respectively) and with more complex measurements of reading competence of the OECD Programme for International Student Assessment study (PISA; preliminary version of SLS 5-8: *r* = 0.64; Wimmer and Mayringer, 2014). Moreover, adolescents with low test scores in sentences tasks similar to those used in the children’s SLS showed a higher number of fixations and also a longer gaze duration (Hawelka et al., 2010).

While English reading research and diagnostic assessment is rather privileged to further evaluate reading performance over the life span with tests based on sentence and text reading, no such thing has been established for German (older) adults. This is rather surprising. Even though there is evidence that core reading skills are preserved, the process of reading, the plasticity of its associated neural networks as well as its supporting cognitive functions undergo at least some developmental changes across the entire life span (e.g., Allen et al., 1991; Spieler and Balota, 2000; Hedden and Gabrieli, 2004; Rönnlund et al., 2005; Madden et al., 2007; Stine-Morrow et al., 2008; Froehlich et al., 2016; Froehlich et al., 2018). Moreover, as German and English differ with respect to the depth of their orthographies (e.g., Ziegler and Goswami, 2005), one cannot not simply transfer results of English readers to German readers as has been shown for both children and adults (e.g., Frith et al., 1998; Landerl, 2000; Ziegler et al., 2001; Rau et al., 2015; Oganian et al., 2016). Nonetheless, at the single word level, the age of an adult proficient reader seems to not affect orthographic/lexical and lexico-semantic processing in both orthographies (e.g., Daselaar et al., 2003; Ratcliff et al., 2004; Spaniol et al., 2006; Cohen-Shikora and Balota, 2016; Froehlich and Jacobs, 2016; Froehlich et al., 2016) while sublexical and phonological processing show age-related alterations (e.g., Allen et al., 1991; Madden et al., 2007; Froehlich and Jacobs, 2016; Froehlich et al., 2016). At the sentence and text level, age-related decrements have primarily been reported for surface and discourse processing whereas efficient processing of the situation model seems to be preserved (Radvansky and Dijkstra, 2007). Especially older adults seem to profit from contextual cues in longer texts, however, compared to younger adults, they need to allocate more resources during reading to ensure understanding (e.g., Stine-Morrow et al., 2008). As the vast majority of sentence and text related studies have been conducted with English younger and older participants, it still remains unknown whether these results are applicable to German participants. To our knowledge, the only studies investigating age-related differences in sentence reading in German adults focused primarily on eye tracking measures (Kliegl et al., 2004) or on syntactic complexity (Junker, 2005) underscoring the need for further research within this specific area.

### The Present Study

The overall aim of this study is to introduce a standardized sentence-based screening procedure, the SLS-Berlin, to reliably capture reading proficiency across the life span and thus foster research in that area. For this purpose, we adapted 77 sentences from the speeded sentence reading test (cf. Bergmann and Wimmer, 2008; Hawelka et al., 2010; Wimmer et al., 2010) for which plausibility judgments had to be made. The resulting SLS-Berlin score, i.e., the sum of correctly answered sentences within a 3-min time frame, then indicates the level of reading proficiency which can be compared to an age-matched norming sample. The 3-min cutoff was chosen in accordance with the children’s version of the SLS (Mayringer and Wimmer, 2003; Auer et al., 2005; Wimmer and Mayringer, 2014).

Word-based measures of reading assessment often focus on reading speed and/or accuracy. These measures are somewhat narrow in their scope and underrepresent the construct of reading (Cunningham, 2001; Kuhn and Stahl, 2003). Given the central intermediate character of sentences (cf. see section “Reading Words, Sentences, Texts”), sentences based measures appear to be an ecologically more valid indicator for reading proficiency. Moreover, by providing norms based on a large age diverse sample (ages: 16–88 years, *N* = 2,148), it is not only possible to compare and/or select participants according to their rank in reading proficiency within one cohort but also across cohorts. The latter is especially important as speeded measures usually penalize increasing age (cf. Salthouse, 1996; Stine-Morrow et al., 2008). The SLS-Berlin can be administered to both individuals as well as groups, is easy to implement and to analyze.

Additionally, we aimed at establishing convergent validity for the SLS-Berlin. To assess the test’s ability to capture basic early processes of reading proficiency, participants had to first read words or pseudowords. Secondly, to validate the SLS-Berlin’s potential of assessing more complex, i.e., later, processes of reading proficiency, participants had to read texts with their eye-movements being recorded. Eye-tracking, being rather elaborate and time consuming for a simple screening procedure, is an otherwise excellent method to investigate individual differences in reading performance, including developing and aging readers (Schroeder et al., 2015). Whereas measures such as first fixation duration (FFD) are thought to reflect early processes related to visual word recognition and orthographic/lexical access, measures such as total reading time (TRT) are considered to capture later processes associated with comprehension (e.g., Hyönä et al., 2003; Radach and Kennedy, 2004).

Finally, as we collected data from 1,493 persons being at least 60 years or older, we also expect to shed more light on the development of written language processing during later and late adulthood in German given the sparsity of available data, especially when it comes to sentence and text reading, i.e., reading in an ecologically valid setting.

To meet our objectives, in Study 1, we describe the sentence characteristics for all 77 sentences included in the SLS-Berlin as well as the characteristics of the norm sample. We report the distribution of the SLS-Berlin scores for the entire sample and the dependencies of the score from the sample characteristics. Additionally, we present how sentence characteristics relate to the response times of all sentences. Finally, Study 2 was conducted to further validate the SLS-Berlin. To do so, we compared the SLS-Berlin test scores of a new sample with two single (pseudo)word reading tests, one processing speed and attention test and eye-movements during text-reading.

## Study 1: the SLS-Berlin

### Methods

#### SLS-Berlin

Contrary to the children’s version of the SLS, the SLS-Berlin is a computer-based screening test. After a short adaptation phase, participants must read all 77 sentences which increase in their complexity. Participants are instructed to verify the content of each of the sentences by deciding whether it violates basic world knowledge. Participants are instructed to make the plausibility judgments for all sentences as quickly and as accurately as possible. In accordance with the children’s version of the SLS, the final SLS-Berlin score is derived from the number of correctly judged sentences within a 3-min time frame. To deemphasize the focus on processing speed, the 3-min cut-off is not mentioned, and participants are not interrupted while reading and judging the 77 test sentences. On average, it takes participants approximately 7 min to complete the test.

#### Stimuli

The 77 sentences presented within the SLS-Berlin were taken from a pool of 154 sentences for an adult paper-pencil version (Bergmann and Wimmer, 2008; Hawelka et al., 2010; Wimmer et al., 2010) of the SLS 1-4 (Wimmer and Mayringer, 2014). To minimize shallow processing and skim reading all sentences included at least one long word (with more than six letters). Sentences contained either confirmations or violations of basic world knowledge (e.g., “*For safety reasons, smoking is prohibited at petrol stations*” and “*With a scale, the height of a person is measured,”* respectively). Correct (confirmations) and incorrect (violations) sentences did not differ with respect to sentence length, number of punctuation marks and number of long words. In total, we selected 36 correct and 41 incorrect easy to understand sentences of varying length (ranging from 4 to 18 words; see Supplementary Table 1). Table 1 reports sentence characteristics associated with sentence length, such as Number of all Characters, Number of Syllables, Number of Words, and Number of Long Words (with more than six letters) as well as sentence characteristics typically associated with syntax, such as Number of Nouns, Number of Punctuation Marks and the German adaptation of the Flesch’s Reading Ease Index (Amstad, 1978). Also reported are the results of the *t*-tests comparing sentence characteristics between correct and incorrect sentences.

**TABLE 1:** Descriptive statistics of different sentences characteristics for all 77 SLS-Berlin sentences and differences between correct and incorrect sentences.

						**Difference between correct and incorrect sentences**	
	**Mean**	**SD**	**Median**	**Minimum**	**Maximum**	***t*(76)**	**p**
Number of characters (including blanks)	85.97	18.06	83.00	40.00	139.00	< |1|	−
Number of syllables	25.38	5.72	25.00	10.00	40.00	< |1|	−
Number of words	10.96	2.91	11.00	4.00	18.00	< |1|	−
Number of long words (>6 letters)	4.27	1.46	4.00	1.00	10.00	< |1|	−
Number of nouns	3.01	0.90	3.00	1.00	5.00	−1.13	0.26
Number of punctuation marks	1.64	0.76	1.00	1.00	4.00	< |1|	−
Flesch	28.36	32.73	33.06	–72.63	86.00	< |1|	−
Complexity rating^*^	2.29	0.50	2.21	1.00	3.68	< |1|	−
Point of decision^*^	92.83	9.42	95.79	58.33	100.00	3.60	<0.0001

^*^Complexity was rated by 19 participants (different from the norm sample) on a five-point scale ranging from 1 to 5. They also marked the point of semantic decision for all 77 sentences.

**FIGURE 1:**
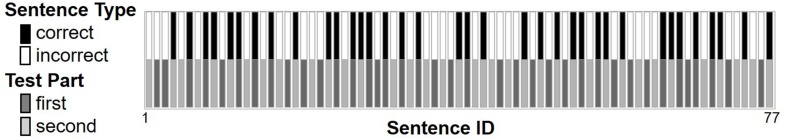
Distribution of correct and incorrect sentences (upper part) and split of all 77 sentences into first and second test part to calculate split-half reliability (lower part).

For the final version of the SLS-Berlin, sentences were ordered in a fixed sequence of correct and incorrect sentences so that no more than three sentences of one type were presented in a row (see Figure 1, upper section). Sentence order followed the principle of increasing complexity with sentences containing fewer and shorter words being shown more to the beginning and sentences with more and longer words closer to the end of the test (see Table 2 for examples of simple and more complex correct and incorrect sentences).

**TABLE 2:** Easy and complex examples for correct and incorrect sentences used in the SLS-Berlin.

**Sentence ID**	**Condition**	**Sentences [English Translations]**
1	Incorrect	Ein Nashorn ist ein Blechblasinstrument. [A rhinoceros is a brass instrument.]
4	Correct	Ein Mobiltelefon ist sehr praktisch, wenn man unterwegstelefonieren will. [A mobile phone is very useful to do a phone call on the road.]
76	Incorrect	Bei einem Symposium folgt dem Vortrag eines Referenten über kontroverseInhaltegewöhnlich ein Wettrennen. [At a symposium a speaker’s presentation about controversial contents is usually followed by a race.]
77	Correct	Bei sportlichenAktivitätenempfehlen sich Kleidungsstücke aus funktionellenMaterialien, die schnell trocknen und besonders reißfest sind. [When doing sportive activities, it is sensible to wear clothes of functional materials, which easily dry and are extremely tear-resistant].

*Underlined words contain more than six letters.*

The principle of increasing complexity was tested in a prestudy. Here, 19 native German readers (*M* = 22.2 years, *SD* = 1.92, range: 19–26, 14 females) made plausibility judgments on each of the 77 sentences, marked the Point of Decision (word at which plausibility judgment was possible), and rated the Sentence Complexity on a scale ranging from 1 (simple) to 5 (complex). Mean ratings for Sentence Complexity ranged from 1 (sentence 1) to 3.68 (sentence 69, see also Supplementary Table 1). The Mean Point of Decision (reported in percent) occurs rather late in the sentence, i.e., after reading more than 90% of the sentence (*M* = 92.8%, *SD* = 9.43%).

The intended increasing complexity was verified by a regression analysis^1^ predicting Sentence Complexity ratings by sentence position within the test (Sentence ID), which identified a positive cubic relationship: adjusted (adj.) *R^2^* = 0.51, *F*(1,74) = 27.3, *p* < 0.001.

To further describe the sentences used in the SLS-Berlin, we conducted regression analyses (cf., see section “Study 1-Data Analysis”) with Sentence ID as a predictor for all sentence characteristics. The results are reported in Table 3. Significant positive cubic relationships were identified for Number of all Characters, Number of Syllables and Number of Words [all *adj. R^2^s* > 0.27, all *F*s(1,74) > 10.5, all *p*s < 0.001]. For Number of Nouns, Number of Long Words and the Flesch Index significant linear relationships were observed [all *adj. R^2^s* > 0.15, all *F*s(1,74) > 13.8, all *p*s < 0.001]. These significant results all confirm the (non-linearly) increasing complexity of the sentences. Only for one sentence characteristic, Mean Point of Decision (in percent), no significant relationship with Sentence ID was observed (*F* < 1). This was also the only variable for which the comparison for correct and incorrect sentences indicates a significant difference [*t*(76) = 3.60, *p* < 0.001, *d* = 0.82]. The Mean Point of Decision (in percent) for incorrect sentences (*M* = 89.6%, *SD* = 11.5%) occurs earlier than for correct sentences (*M* = 96.7%, *SD* = 3.63%). However, most importantly, the response times of incorrect vs. correct sentences did not differ from each other within the norm and the subsamples (see the following section; all *t*s < |0.34|, all *p*s > 0.73).

**TABLE 3:** Model parameters and summaries of linear and polynomial regression analyses of Sentence ID predicting SLS-Berlin sentence characteristics.

	**Model parameters**				**Model summary**		
	**Estimate**	***SE***	***t*-value**	***p*-value**	***adj. R*^2^**	***F-*value**	***p*-value**
*Number of characters*							
Linear	2.27	0.57	4.01	<0.001	−	−	−
Squared	–0.06	0.02	–3.82	<0.001	−	−	−
Cubic	0.0006	0.0001	4.33	<0.001	0.65	47.77	<0.001
*Number of syllables*							
Linear	0.55	0.19	2.82	0.006	−	−	−
Squared	–0.01	0.006	–2.57	0.010	−	−	−
Cubic	0.0001	0.00005	3.0	0.004	0.59	36.99	<0.001
*Number of words*							
Linear	0.41	0.12	3.15	0.002	−	−	−
Squared	–0.01	0.004	–3.26	0.002	−	−	−
Cubic	0.0001	0.00003	3.51	<0.001	0.27	10.46	<0.001
*Number of long words^a^ (>6 letters)*							
Linear	0.03	0.006	4.79	<0.001	0.23	22.99	<0.001
Squared	−	−	−	−	−	−	−
Cubic	−	−	−	−	−	−	−
*Number of nouns*							
Linear	0.02	0.004	4.59	<0.001	0.21	21.05	<0.001
Squared	−	−	−	−	−	−	−
Cubic	−	−	−	−	−	−	−
*Number of punctuation marks*							
Linear	−	−	−	−		<1	*n*.*s*.
Squared	−	−	−	−			
Cubic	−	−	−	−			
*Flesch*^b^							
Linear	–0.52	0.14	–3.72	<0.001	0.15	13.81	<0.001
Squared	−	−	−	−	−	−	−
Cubic	−	−	−	−	−	−	−
Complexity rating^*^							
Linear	0.06	0.019	3.44	<0.001	−	−	−
Squared	–0.002	0.0006	–3.34	0.001	−	−	−
Cubic	0.00002	0.000005	3.71	<0.001	0.51	27.35	<0.001
Point of decision^*c^							
Linear	−	−	−	−		<1	*n*.*s*.
Squared	−	−	−	−			
Cubic	−	−	−	−			

^*^In a prestudy 19 participants (different from the norm sample) rated the complexity (on a five-point scale ranging from one to five) and marked the point of semantic decision for all 77 sentences. ^*a*^Best model fit after excluding influential cases (i.e., sentence 77) due to large residuals (< ± 3). ^*b*^Best model fit after excluding influential cases (i.e., sentences 8 and 28) due to large residuals (< ± 3). ^*c*^Best model fit after excluding influential cases (i.e., sentences 10 and 33) due to large residuals (< ± 3).

#### Norm Sample

We collected data from 2,174 native German speaking adults with age ranging from 16 to 88 years. Twenty-three participants had to be excluded as they obtained negative SLS-Berlin scores (i.e., they classified more sentences incorrectly than correctly within 3 min reading time). Three additional participants were removed from all further analyses due to technical difficulties while recording their data. In all, the norm sample consists of data from 2,148 adult participants (see Table 4 for sample characteristics).

**TABLE 4:** Sample sizes and characteristics of norm sample and the two subsamples of younger adults (<60) and older adults (> = 60).

	**Sample**		
	**All**	**Younger adults (<60)**	**Older adults (> = 60)**
*N (%)*	2.148	655 (30.5)	1.493 (69.5)
Age (years)^*^	58.0 (19.0)	30.4 (7.0)	70.1 (3.9)
Age range^*^	16–88	16–59	60–88
% female	52.6	56.2	51.0
Education (years)^*^	14.6 (3.0)	15.4 (2.8)	14.2 (2.9)
SLS-Berlin^*^	55.6 (11.3)	59.7 (11.0)	53.9 (11.0)

^*^Standard deviations are reported in brackets.

As most research in adult reading is conducted with either younger (ages: 20 to 35) and/or older readers (60 years and older), we focused on these age groups while collecting norm data. However, to provide a norm sample which covers a wide range of the adult life span, we also present data for ages 36 to 59 (see Supplementary Table 2 for norm table). More specifically, we report analyses with respect to the entire norm sample [*N* = 2,148, *M_age_* = 58.9 years, *SD_age_* = 19.0, 1,130 females (52.6%), *M_*education*_* = 14.6 years, *SD*_*education*_ = 2.97] as well as for two subsamples, i.e., for all participants being younger than 60 years of age [subsample younger adults; *N* = 655, *M_*age*_* = 30.4 years, *SD_age_* = 7.01, range: 16–59, 368 females (56.2%)] and those being 60 or above [subsample older adults; *N* = 1,493, *M_*age*_* = 70.1 years, *SD_age_* = 3.93, range: 60–88, 762 females (51.0%)]. The subsamples differed with respect to years of formal education with the younger subsample yielding more years of formal education (*M*_*education*_ = 15.4, *SD_*education*_* = 2.98) than did the older subsample (*M*_*education*_ = 14.2, *SD_*education*_* = 2.89), *t*(1079.1) = 8.06, *p* < 0.001, *d* = 0.41.

All participants had normal or to corrected-to-normal vision, reported no history of language impairment, neurological disease, psychiatric disorders or a history of head injuries. Prior to testing, participants gave written informed consent. Most participants received financial compensation, a minority within the younger subsample obtained course credit for their participation. The study was approved by the Ethics Committee of the Max Planck Institute for Human Development, Berlin (MPIB) and by that of the Freie Universität Berlin (FU Berlin).

#### Procedure

Test sessions took place at the FU Berlin and at the Charité Campus, Berlin between the years 2013 and 2017. The 1,884 subjects tested at the Charité Campus were part of the Berlin Aging Study II cohort (BASE-II: Bertram et al., 2014; Gerstorf et al., 2016) and participated in groups of up to six individuals. At the FU Berlin, data from 264 subjects were collected in individual test sessions. At both locations, additional tests were conducted, however, the SLS-Berlin was always the first part of the experimental session and identical in its procedure.

Contrary to the children’s version of the SLS, the SLS-Berlin was run on a computer while using Objective-C (MPIB) and Python 2.7 (FU). Ten training sentences and the 77 test sentences were shown individually one after another at the center of a computer screen (left aligned and single spaced) in 32 pt Helvetica using black font on white background. Prior the presentation of each sentence a fixation cross appeared at the middle of the screen for 1500 ms, followed by the sentence. Short sentences were presented in one line, longer sentences in two or three lines. Participants were asked to make plausibility judgments as quickly and as accurately as possible after reading each of the sentences. Responses were recorded with the help of a button box or a standard keyboard using the index and middle finger of the left hand. By demanding only little motor movements, the SLS-Berlin is well suited to be used in MRT and EEG settings. Feedback on responses was only given during training trials. The instruction did not stress the time dependency of the test. Contrary to the paper pencil versions for children and adults, the 3-min cut-off for calculating the final test score was not mentioned and participants were not interrupted while reading and judging the 77 test sentences. Overall, the completion of the SLS-Berlin took approximately 7 min. The final SLS-Berlin score of each participant was calculated by adding the number of correctly answered sentences within a 3-min time window of pure reading time (i.e., omitting the times of fixation crosses).

#### Data Analysis

Analyses of the SLS-Berlin scores and the response times were conducted with R version 3.4.3 (R Core Team, 2017). All analyses were performed for the whole norm sample as well as for the two subsamples. In a first step, we assessed the consistency of the test by calculating the split-half reliability. For this purpose, the 77 sentences of the SLS-Berlin were split into two parts with the number of correct and incorrect sentences being roughly the same (part 1 18 correct and 21 incorrect, part 2: 18 correct and 20 incorrect). We also controlled for mean Sentence IDs (part 1: 39.5; part 2: 38.4; cf. Figure 1, lower section) given their predictive power for the other item characteristics. We then obtained the number of correctly answered sentences within 1.5 min per subject to calculate the SLS-Berlin scores for both parts of the test and correlated the results using the Spearman-Brown prophecy formula (Brown, 1910; Spearman, 1910).

In a second step, we analyzed the relationship between the SLS-Berlin score and sample characteristics (i.e., Gender, Age, and Education; see Table 5) as well as assessed the influence of the above described sentence characteristics on the response times for the sentences (see Tables 6**–**8). To do so, we used only correctly classified sentences (data loss of 4.73%). Outliers based on response times were excluded by estimating a linear mixed-effects model with random intercepts for participants and items using the lme4 package (version 1.1-15; Bates et al., 2015). Response times with standardized residuals larger than ±3 were removed from the data set (data loss of 1.48%).

**TABLE 5:** Model parameters and summaries of linear and polynomial regression analyses of SLS-Berlin scores predicted by Age and Education for all participants and the two subsamples of younger (<60 years) and older adults (> = 60 years).

	**Model parameters**				**Model summary**		
	**Estimate**	***SE***	***t*-value**	***p*-value**	***adj. R*^2^**	***F-*value**	***p*-value**
*Age – all^a^*							
Linear	0.48	0.12	3.96	<0.001	−	−	−
Quadratic	–0.007	0.001	–5.21	<0.001	0.07/0.089^*^	87.5	<0.001
Cubic	−	−	−	−	−	−	−
*Age – younger adults*							
Linear	–0.00	0.06	<1	0.96	0.00/0.00^*^	<1	0.96
Quadratic	−	−	−	−	−	−	−
Cubic	−	−	−	−	−	−	−
*Age – older adults^a^*							
Linear	–3.93	1.68	–2.34	0.02	−	−	−
Quadratic	0.02	0.01	2.05	0.04	0.03/0.025^*^	26.15	<0.001
Cubic	−	−	−	−	−	−	−
*Education – all*							
Linear	12.09	3.41	3.55	<0.001	−	−	−
Quadratic	–0.63	0.21	–2.94	<0.01	−	−	−
Cubic	0.01	0.004	2.49	0.012	0.06/0.079^*^	36.59	<0.001
*Education – younger adults*							
Linear	3.40	1.20	2.84	0.005	−	−	−
Quadratic	–0.09	0.04	–2.52	0.012	0.02/0.03^*^	6.85	0.0011
Cubic	−	−	−	−	−	−	−
*Education – older adults*							
Linear	4.16	1.33	3.13	0.002	−	−	−
Quadratic	–0.12	0.05	–2.54	0.011	0.05/0.05^*^	32.27	<0.001
Cubic	−	−	−	−	−	−	−

All models were checked for influential data points and outliers. ^*a*^Best model fit after excluding influential cases (i.e., one subject because of large residuals over ±3.0). ^*^Adjusted *R*^2^ after cross-validation.

**TABLE 6:** Model parameters and summaries of linear and polynomial regression analyses of SLS-Berlin sentence characteristics predicting response times for the entire norm sample.

	**Model parameters**				**Model summary**		
	**Estimate**	***SE***	***t*-value**	***p*-value**	***adj. R*^2^**	***F-*value**	***p*-value**
*Sentence ID*							
Linear	56.7	14.2	3.99	<0.001	−	−	−
Quadratic	–1.59	0.42	–3.77	<0.001	−	−	−
Cubic	0.02	0.00	4.56	<0.001	0.780/0.778^*^	90.6	<0.001
*Number of characters*^a^							
Linear	–19.7	17.1	–1.15	0.25	−	−	−
Quadratic	0.26	0.09	2.81	<0.01	0.728/0.725^*^	100.0	<0.001
Cubic	−	−	−	−	−	−	−
*Number of syllables*^b^							
Linear	–58.5	56.3	–1.04	0.30	−	−	−
Quadratic	2.55	1.02	2.49	<0.05	0.648/0.647^*^	70.0	<0.001
Cubic	−	−	−	−	−	−	−
*Number of words*							
Linear	–167.0	97.0	–1.72	0.09	−	−	−
Quadratic	13.3	4.25	3.14	<0.01	0.510/0.521^*^	40.6	<0.001
Cubic	−	−	−	−	−	−	−
*Number of long words* (> 6 letters)							
Linear	242.3	35.8	6.78	<0.001	0.372/0.371^*^	45.9	<0.001
Quadratic	−	−	−	−	−	−	−
Cubic	−	−	−	−	−	−	−
*Number of nouns*							
Linear	334.6	62.9	5.32	<0.001	0.264/0.264^*^	28.3	<0.001
Quadratic	−	−	−	−	−	−	−
Cubic	−	−	−	−	−	−	−
*Flesch*							
Linear	−	−	−	−	−	<1	*n*.*s*.
Quadratic	−	−	−	−	−	−	−
Cubic	−	−	−	−	−	−	−
*Complexity ratings^c^*							
Linear	990.4	63.9	15.5	<0.001	0.759/0.766^*^	240.2	<0.001
Quadratic	−	−	−	−	−	−	−
Cubic	−	−	−	−	−	−	−
*Point of decision^c^*							
Linear	–152.1	113.6	–1.34	0.18	−	−	−
Quadratic	13.4	5.33	2.52	<0.05	0.462/0.468^*^	33.6	<0.001
Cubic	−	−	−	−	−	−	−

All models were checked for influential data points and outliers. ^*a*^Best model fit after excluding influential cases (i.e., sentences 1 and 77) due to a Cook’s distance > 1 and high leverage. ^*b*^Best model fit after excluding influential cases (i.e., sentence 1) due to a Cook’s distance > 1 and high leverage. ^*c*^In a prestudy 19 participants (different from the norm sample) rated the complexity (on a five-point scale ranging from one to five) and marked the point of semantic decision for all 77 sentences. ^*^Adjusted *R*^2^ after cross-validation.

**TABLE 7:** Model parameters and summaries of linear and polynomial regression analyses of SLS-Berlin sentence characteristics predicting response times for the subsample of younger adults (<60 years).

	**Model parameters**				**Model summary**		
	**Estimate**	***SE***	***t*-value**	***p*-value**	***adj. R*^2^**	***F-*value**	***p*-value**
*Sentence ID*							
Linear	65.08	13.5	4.83	<0.001	−	−	−
Quadratic	–1.70	0.40	–4.25	<0.001	−	−	−
Cubic	0.02	0.00	4.91	<0.001	0.816/0.820^*^	113.3	<0.001
*Number of characters*^a^							
Linear	–265.9	107.7	–2.47	<0.05	−	−	−
Quadratic	3.09	1.16	2.68	<0.01	−	−	−
Cubic	–0.01	0.00	–2.62	<0.05	0.700/0.687^*^	59.2	<0.001
*Number of syllables*^a^							
Linear	82.5	7.64	10.8	<0.001	0.606/0.633^*^	116.5	<0.001
Quadratic	−	−	−	−	−	−	−
Cubic	−	−	−	−	−	−	−
*Number of words*							
Linear	–167.4	108.5	–1.54	0.12	−	−	−
Quadratic	13.1	4.75	4.75	<0.01	0.432/0.439^*^	29.9	<0.001
Cubic	−	−	−	−	−	−	−
*Number of long words* (>6 letters)							
Linear	243.2	37.9	6.42	<0.001	0.346/0.343^*^	41.2	<0.001
Quadratic	−	−	−	−	−	−	−
Cubic	−	−	−	−	−	−	−
*Number of nouns*							
Linear	364.7	64.1	5.69	<0.001	0.292/0.280^*^	32.4	<0.001
Quadratic	−	−	−	−	−	−	−
Cubic	−	−	−	−	−	−	−
*Flesch*							
Linear	–2.15	2.08	–1.03	0.31	0.001/−0.002^*^	1.06	0.31
Quadratic	−	−	−	−	−	−	−
Cubic	−	−	−	−	−	−	−
*Complexity ratings^b^*							
Linear	994.0	73.1	13.6	<0.001	0.708/0.704^*^	184.9	<0.001
Quadratic	−	−	−	−	−	−	−
Cubic	−	−	−	−	−	−	−
*Point of decision^b^*							
Linear	–134.3	123.0	–1.09	0.28	−	−	−
Quadratic	12.5	5.77	2.17	<0.05	0.415/0.401^*^	28.0	<0.001
Cubic	−	−	−	−	−	−	−

All models were checked for influential data points and outliers. ^*a*^Best model fit after excluding influential cases (i.e., sentence 1) due to a Cook’s distance > 1 and high leverage. ^*b*^In a prestudy 19 participants rated the complexity (on a five-point scale ranging from one to five) and marked the point of semantic decision for all 77 sentences. ^*^Adjusted *R*^2^ after cross-validation.

**TABLE 8:** Model parameters and summaries of linear and polynomial regression analyses of SLS-Berlin sentence characteristics predicting response times for the subsample of older adults (> = 60 years).

	**Model parameters**				**Model summary**		
	**Estimate**	***SE***	***t*-value**	***p*-value**	***adj. R*^2^**	***F-*value**	***p*-value**
*Sentence ID*							
Linear	52.8	15.4	3.44	<0.001	−	−	−
Quadratic	–1.54	0.46	–3.37	<0.01	−	−	−
Cubic	0.02	0.00	4.16	<0.001	0.741/0.744^*^	73.3	<0.001
*Number of characters*^a^							
Linear	–27.2	17.0	–1.60	0.11	−	−	−
Quadratic	0.30	0.09	3.27	<0.01	0.733/0.735^*^	102.5	<0.001
Cubic	−	−	−	−	−	−	−
*Number of syllables*							
Linear	–13.6	44.0	–0.31	0.76	−	−	−
Quadratic	1.76	0.82	2.15	<0.05	0.651/0.624^*^	72.0	<0.001
Cubic	−	−	−	−	−	−	−
*Number of words* (>6 letters)							
Linear	546.2	359.2	1.52	0.13	−	−	−
Quadratic	–55.4	33.7	–1.64	0.10	−	−	−
Cubic	2.08	1.00	2.06	<0.05	0.555/0.565^*^	32.6	<0.001
*Number of long words*^b^							
Linear	–102.5	182.6	–0.56	0.58	−	−	−
Quadratic	40.6	20.1	2.02	<0.05	0.375/0.376^*^	23.5	<0.001
Cubic	−	−	−	−	−	−	−
*Number of nouns*							
Linear	320.1	63.6	5.03	<0.001	0.243/0.244^*^	25.3	<0.001
Quadratic	−	−	−	−	−	−	−
Cubic	−	−	−	−	−	−	−
*Flesch*							
Linear	–0.56	2.01	–0.28	0.78	−0.012/−0.026^*^	0.08	0.78
Quadratic	−	−	−	−	−	−	−
Cubic	−	−	−	−	−	−	−
*Complexity ratings^c^*							
Linear	57.0	419.8	0.14	0.89	−	−	−
Quadratic	190.1	84.6	2.25	<0.05	0.776/0.779^*^	133.0	<0.001
Cubic	−	−	−	−	−	−	−
*Point of decision^c^*							
Linear	–165.9	112.0	–1.48	0.14	−	−	−
Quadratic	14.1	5.25	2.68	<0.01	0.473/0.474^*^	35.1	<0.001
Cubic	−	−	−	−	−	−	−

All models were checked for influential data points and outliers. ^*a*^Best model fit after excluding influential cases (i.e., sentences 1 and 77) due to a Cook’s distance > 1 and high leverage. ^*b*^Best model fit after excluding influential cases (i.e., sentence 77) due to a Cook’s distance > 1 and high leverage. ^*c*^In a prestudy 19 participants (different from the norm sample) rated the complexity (on a five-point scale ranging from one to five) and marked the point of semantic decision for all 77 sentences. ^*^Adjusted *R*^2^ after cross-validation.

To assess the model fit of the regression analyses and to test the accuracy of the predictions, we randomly assigned 70% of the sample data to a training group. The remaining 30% were assigned to a test group. Neither the training nor the test group differed in terms of mean response times, age, gender distribution, years of education and mean SLS-Berlin score from the complete sample as well as from each other (all *p*’s > 0.20). We conducted linear, quadratic and cubic regression analyses for each of the sentence characteristics. All regression models were checked for influential cases by inspecting Cook’s distance (Cook, 1977), the models’ standardized residuals as well as the data’s leverage. Observations were removed if Cook’s distance was larger than 1 (Pardoe, 2012), if standardized residuals were larger than ±3 or if they had a leverage above 3^*^(k+1)/n with *k* being the number of predictors in the model and *n* the sample size. Finally, we identified the best fitting model for each sentence characteristic and fitted the winning model on the remaining 30% of the population to compare the predicted with the observed values. If these values turned out to be comparable, this regression model was applied to the entire population. Model parameters and summaries as well as results from the cross-validation procedure are displayed in Tables 6**–**8.

### Results

#### Split-Half Reliability

The internal consistency of the SLS-Berlin is very high with a split-half reliability for the test scores of *r_*All*_* = 0.91, *t*(2146) = 101.1, *p* < 0.001 for the entire norm sample. Separate analyses showed a slightly higher reliability for the younger compared to the older subsample, *r*_*Y*_ = 0.93, *t*(653) = 66.0, *p* < 0.001 and *r*_*O*_ = 0.89, *t*(1,491) = 73.5, *p* < 0.001. The comparison of both reliability coefficients with Fischer’s z by using the *cocor* module (Diedenhofen and Musch, 2015) indicated statistical significance (*z* = 5.03, *p* < 0.001, CI [0.02, 0.05]). Correcting for original test length using the Spearman-Brown formula, reliabilities increased to *r*_*All*_ = 0.95 for the entire norm sample and to *r*_*Y*_ = 0.97 and *r*_*O*_ = 0.94 for the younger and older subsamples, respectively. All coefficients indicate a high test reliability (cf. Flom et al., 2012).

#### Distribution of SLS-Berlin Scores and Predictions by Sample Characteristics

**FIGURE 2:**
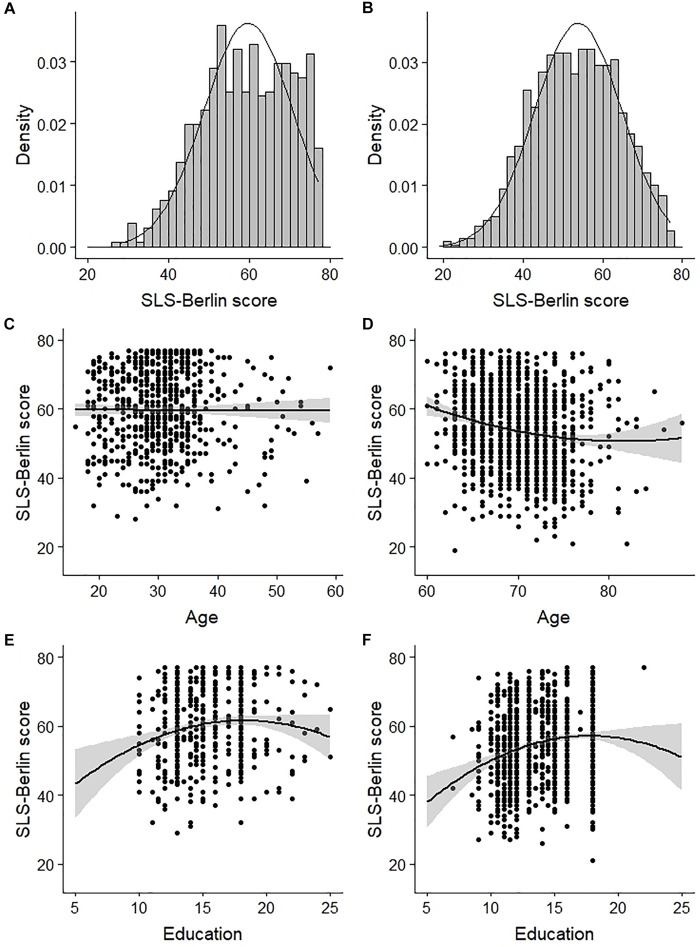
Distribution of the SLS-Berlin scores in the two subsamples of younger **(A)** and older adults **(B)** as well as the relationship of Age and Education on the SLS-Berlin score for the subsamples of younger **(C,E)** and older adults **(D,F)**.

Figures 2A,B shows the distribution of SLS-Berlin scores for the younger and older subsample. 3.21% of the younger and 0.54% of the older adults scored the highest possible value (77) leading to slightly left-skewed distributions. We observed no gender differences, neither in the entire norm sample (*t* < 1) nor in one of the two subsamples (both *t*s ≤ 1), however, both Age and Education significantly predicted SLS-Berlin scores within the norm sample (both *adj. R*^2^s < 0.07, *p*s < 0.001; see Table 5). For Age, we observed a quadratic, for Education a cubic relationship. Separate analyses for the two subsamples indicated no effect of Age on SLS-Berlin scores for younger adults (*F* < 1), yet a significant influence was observed for the older subsample (*adj. R^2^* = 0.03, *p* < 0.001; see Figures 2C–F and Table 5). The quadratic nature of this relationship resulted from a steeper decrease starting around age of 60, which flattened around the age of 70. For Education, we observed small, albeit significant, quadratic effects for both subsamples. It was slightly stronger for older (*adj. R*^2^ = 0.05, *p* < 0.001) compared to younger adults (*adj. R^2^* = 0.03, *p* = 0.001). Similar low adjusted *R*^2^s from the cross-validation procedure underpinned the generally small effects of Age and Education on SLS-Berlin scores.

#### Regression Analysis of Response Times

**FIGURE 3:**
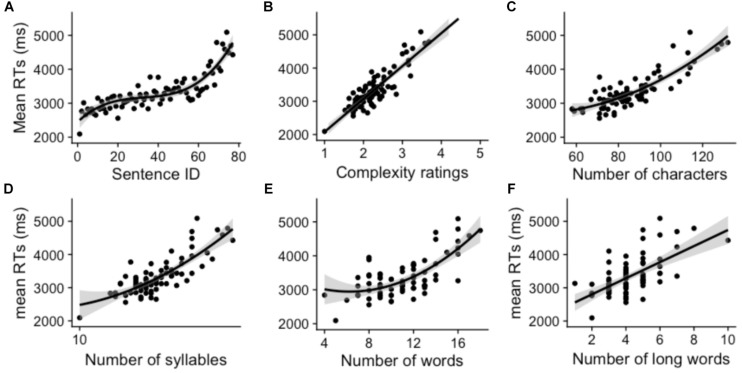
Relationships between sentence characteristics Sentence ID **(A)**, Complexity ratings **(B)**, Number of characters **(C)**, Number of syllables **(D)**, Number of words **(E)**, and Number of long words **(F)** and response times for the test sentences from the SLS-Berlin for the entire norm sample.

Model parameters and summaries of linear and polynomial regressions for the norm sample as well as the two subsamples of younger and older adults are displayed in Tables 6**–**8, respectively. The cross-validation produced very similar *adj. R*^2^ compared to final models calculated for all participants (cf. Table 6), for the younger subsample (cf. Table 7) and the older subsample (cf. Table 8) indicating a very good generalizability of our data. Figure 3 depicts the relationships between six selected sentence characteristics and the average response times for the 77 sentences from the SLS-Berlin.

##### Norm sample

Except for the German adaptation of the Flesch Reading Ease Scores (Amstad, 1978) and the Mean Point of Decision (in percent), all SLS-Berlin sentence characteristics predicted significantly the response times in the norm sample with *adj. R*^2^ ranging from 0.26 to 0.78, all *F*s > 28.3, all *p*s < 0.001. Best model fit (*adj. R*^2^) was obtained for Sentence ID (0.78), followed closely by Complexity Ratings (0.77) and further by Number of Characters (0.73). Number of Syllables showed the fourth best fit (0.65). Word related measures, i.e., Number of Words, Number of Long Words, and Number of Nouns followed suit (0.51, 0.37, and 0.26, respectively). However, the nature of the relationship between sentence characteristics and response times varied with respect to the predictor (see Figure 3). While a linear relationship was observed for Number of Characters, Number of Syllables and Number of Words, a quadratic one was registered for Complexity Ratings, Number of Words and Number of Long Words. Sentence ID produced a cubic model fit.

##### Younger subsample

Results of the younger subsample were very similar to those of the norm sample. Again, the German adaptation of the Flesch Reading Ease Scores and the Mean Point of Decision (in percent) showed no predictive power on response times. All other seven sentence characteristics produced slightly higher *adj. R*^2^ than the norm sample ranging from 0.29 to 0.82, all *F*s > 29.9, all *p*s < 0.001. The order with which significant predictors explained data variance (*adj. R*^2^) was identical to that of the norm sample: Sentence ID (0.82), Complexity Ratings (0.71), Number of Characters (0.70), Number of Syllables (0.61), Number of Words (0.43), Number of Long Words (0.35), and Number of Nouns (0.29).

Comparable to the norm sample, we found linear relationships for Complexity Ratings, Number of Long Words and Number of Nouns. However, with respect to Number of Syllables the linear regression also showed the best model fit (vs. a quadratic one in the norm sample). The only quadratic relationship between predictor and outcome variable was identified for Number of Words (vs. Number of Words, Number of Characters and Numbers of Syllables in the norm sample). Cubic model fits were observed for Sentence ID (as was in the norm sample) and additionally for Number of Characters (vs. a quadratic fit in the norm sample).

##### Older subsample

Like the younger subsample, results of the older subsample resembled those of the norm sample. As before, the German adaptation of the Flesch Reading Ease Scores and the Mean Point of Decision (in percent) did not yield any significant effects while the other seven sentence characteristics produced reliable model fits, with adjusted *R*^2^s ranging from 0.24 to 0.78, all *F*s > 23.5, all *p*s < 0.001. However, different from the norm sample and the younger subsample, the order of the strongest two predictors (*adj. R^2^)* was reversed with Complexity Ratings explaining more variance in response times (0.78) than did Sentence ID (0.74). The order of the other predictors, e.g., Number of Characters (0.73), Number of Syllables (0.65), Number of Words (0.56), Number of Long Words (0.38), and Number of Nouns (0.24) was yet again comparable to that of the norm sample and the younger subsample.

The only linear relationship was identified for Number of Nouns (as was in the norm sample and the younger subsample). Complexity Ratings, Number of Characters and Number of Syllables all showed quadratic fits (the latter two in accordance with the norm sample, while the first being linear in the norm sample). Sentence ID and Number of Words yielded a cubic relationship between predictor and outcome variables in the older subsample (the first in accordance with the norm sample while the latter being quadratic in the norm sample).

### Discussion

We collected data from > 2,000 participants including a group of younger (below 60 years of age) and a group of older adults (above 60). The split-half reliabilities for the entire norm sample as well as for the two subsamples indicated a high consistency of the SLS-Berlin for all investigated age groups.

Contrary to the children’s version of the SLS, which reports slightly higher reading scores for girls than for boys (cf. Wimmer and Mayringer, 2014), the SLS-Berlin is independent of gender. This is well in line with current results about gender differences in cognitive components of adult reading comprehension. As pointed out by Hannon (2014), quantitative gender differences are mostly marginal, especially in comparison to the more important differences in the predictive powers of specific cognitive components of reading comprehension.

In both the younger and the older subsample, years of formal education had only minor influences on SLS-Berlin scores, i.e., test scores slightly increased with increasing years of education. Age related effects were observed for older adults only. The quadratic relationship indicated a steeper decline at the age of 60, which almost leveled out around 70 years of age. These rather late effects correspond to reported age effects for lexical access during single word recognition (e.g., Cohen-Shikora and Balota, 2016; Froehlich et al., 2016) and building successful situational representations in text comprehension (e.g., Radvansky, 1999; Radvansky and Dijkstra, 2007). A relatively stable performance up to the age of 60 has likewise been described in longitudinal studies for cognitive functions such as verbal ability, verbal (working) memory as well as episodic memory (Hedden and Gabrieli, 2004; Rönnlund et al., 2005) known to contribute to reading performance (DeDe et al., 2004; Stine-Morrow et al., 2008; Frick et al., 2011).

Taken together, the SLS-Berlin is a robust screening test to assess reading proficiency over the entire life span. The absence of gender effects, the minor effects of Education and Age all imply versatile application possibilities in both, research and educational/diagnostic settings. Particularly, the absence of significant age effects in the younger age group below 60, marks the SLS-Berlin as an excellent screening test for research projects focusing on changes in cognitive functioning across the life span.

Our detailed analyses of response times of the test sentences constituting the SLS-Berlin clearly underpin the increasing complexity, i.e., the further the test progresses the more complex the sentences become (cf., see section “Study 1-Stimuli”). This distinguishes the SLS-Berlin from the Woodcock-Johnson Sentence Reading Fluency Task (Schrank et al., 2014). The increasing complexity relates directly to increasing reading times as the regression analyses of Complexity Ratings on response times showed for the entire norm sample as well as for both subsamples. Sentence ID as well as Complexity Ratings were the strongest predictors for reading times. That is not surprising given that Sentence ID is positively confounded with all other item characteristics. The same can be assumed for Complexity Ratings. The cubic relationship between Sentence ID and response times indicated a strong increase in response times, especially within the first 20 and the last 20 sentences. Therefore, we conclude that the SLS-Berlin is not only sensitive to individual differences within poor readers, between poor and good readers but also within good readers. Nevertheless, the sensitivity for the upper performance spectrum of reading proficiency appears to be not as high as for the lower spectrum as indicated by the slightly right-skewed distribution of SLS-Berlin scores.

The reported relationships between sentence characteristics and response times also indicated that neither the Flesch’s Reading Ease Index nor the Mean Point of Decision (in percent) reliably explained variance in response times. As the Flesch’s Reading Ease Index has been developed to particularly assess the difficulty of texts, the former result is not surprising and suggests the evaluation of the complexity of single sentences with the help of rating studies rather than the Flesch’s Reading Ease Index. The finding regarding the Mean Point of Decision (in percent) suggests that participants had read the complete sentences of the SLS-Berlin rather than terminating the reading process early, for instance, before or as soon as a pragmatic violation within the sentence occurred. This underscores particularly well the fact that the SLS-Berlin is sensitive to both early visual processing associated with decoding and later comprehension processes. In order to further explore this conclusion and to fully validate the SLS-Berlin, we compared it to other, already established, measures of reading (ability).

## Study 2: Comparing the Sls-Berlin to Other Reading Measures

Study 2 was conducted to describe the relationship between SLS-Berlin and other reading measures. More importantly, to show that our screening test at the sentence level captures both basic decoding processes such as word recognition and later more complex processes such as meaning comprehension and integration. We therefore compared the SLS-Berlin scores with results from single word and pseudoword reading as well as from tasks focusing on text comprehension. Relating performance in single word processing as well as text reading to the SLS-Berlin captures the two essential foci of empirical research about text reading (Stine-Morrow et al., 2006; De Beni et al., 2007; Radvansky and Dijkstra, 2007; Borella et al., 2011; Froehlich et al., 2018) and the development and changes in reading and language comprehension over the life span (Hannon, 2012; Hannon and Frias, 2012; Froehlich and Jacobs, 2016; Froehlich et al., 2018).

To evaluate the relationship between the SLS-Berlin and measures for word identification, we correlated SLS-Berlin scores with a well-established test for single word and pseudoword reading. Considering that timed reading of unconnected words and pseudowords underrepresent the construct of reading fluency (Cunningham, 2001; Kuhn and Stahl, 2003), we expected strong but not perfect correlations of SLS-Berlin scores with these very basal measures of reading proficiency.

Similar to other standardized tests of reading fluency and proficiency for children and adults (e.g., SLRT-II, SLS 2-9, Sentence Reading Fluency Test from Woodcock-Johnson IV: Tests of Cognitive Abilities), scores of the SLS-Berlin are time dependent. Even though participants are not made aware of the 3-min cutoff, they are instructed to read as fast and as accurately as possible. Given the time dependency of the SLS-Berlin participants with higher processing speed should have an advantage compared to participants with lower processing speed regardless of their reading proficiency. Therefore, we additionally administered a test for visual attention and processing speed. Here, we expected a positive correlation between general processing speed and SLS-Berlin scores. However, this correlation should be noticeably weaker than the correlations between SLS-Berlin scores and the indicators for word/pseudoword reading and text comprehension as the SLS-Berlin was designed to specifically assess reading proficiency without emphasizing the speed component.

To further evaluate the SLS-Berlin’s ability and to capture more complex processes related to comprehension, we also obtained eye-tracking data during text reading as an external criterion (cf. Moore and Gordon, 2015). Eye-tracking is an excellent method to investigate visual and cognitive processing during text reading and comprehension (Adler-Grinberg and Stark, 1978; Duffy, 1992; Rayner et al., 2006). Several studies conducted with both, children and adults, demonstrated that individual differences in reading fluency and proficiency are associated with differences in eye movement patterns (Kemper et al., 2004; Ashby et al., 2005; Hawelka et al., 2010; Kuperman and Van Dyke, 2011). Fast readers compared to slow readers, for example, were found to produce fewer fixations, shorter gaze durations, larger saccades and more skipping when reading single sentences and short texts (Ashby et al., 2005; Thaler et al., 2009; Hawelka et al., 2015; Krieber et al., 2016). Corresponding patterns were found for the differences between dyslexic and normal readers (Hyönä and Olson, 1995; Hutzler and Wimmer, 2004; Hawelka et al., 2010). If, as assumed, the SLS-Berlin score reliably measures individual differences in reading proficiency, it should therefore also reliably predict differences in eye movement patterns observed during text reading. Moreover, eye-tracking research in text comprehension indicates that eye movement measures reflect also higher order comprehension processes to some extent (Duffy, 1992; Rayner et al., 2006). Rayner et al. (2006) assumed, that “higher order comprehension processes can override the normal default situation in which lexical processing is driving the eyes and result in longer fixations or regressions back to earlier parts of the text” (p. 244). To underline the SLS-Berlin’s sensitivity for both early and automatic decoding processes as well as higher order comprehension processes, we analyzed two different eye-tracking parameters, i.e., FFD and TRT. While the former is usually associated with initial visual recognition and lexical access, the latter is related to higher order comprehension processes (see Table 16.4 in Hyönä et al., 2003, and Tables 3, 4 in Boston et al., 2008, for a detailed description of eye movement measures and relevant references). Eye tracking data was obtained while participants read two texts with different levels of complexity. For the less complex text, we assumed the SLS-Berlin to be highly predictive, especially with regards to TRT. However, as higher order comprehension processes associated with reading difficult texts may overwrite basic processes related to reading proficiency (cf. Rayner et al., 2006), we expected less predictive power of SLS-Berlin scores on FFD and TRT in the more complex text.

### Methods

#### Test Materials

##### Word and pseudoword recognition

To measure word and pseudoword recognition, we conducted two subtests of the *Salzburger Lese- und Rechtschreib-Test* [Salzburg Reading and Spelling Test (SLRT II); Moll and Landerl, 2010]. Each subtest contains a word and a pseudoword list with increasing item length and complexity. Participants are asked to read out loud as much items as possible without making pronunciation errors. The final score is the number of correctly read items within 1 min and is separately obtained for words and pseudowords.

##### Visual attention and processing speed

To measure serial allocation of visual attention and discrimination as well as processing speed, we used a language free version (i.e., the ‘Smiley-Test’; cf. Landerl, 2001; Marx et al., 2016) of the *d2-R* test (Brickenkamp et al., 2010). In the original version of the d2-R, participants receive a sheet of paper containing nine lines with the letters d and p. They mark all cases where the letter d is flanked by two quotation marks, all other cases must be ignored. In the language free version, the letters are replaced by line-drawings of happy and unhappy faces. Participants mark the smiley faces flanked by two quotation marks while ignoring the distractors. Each of the nine lines comprises 47 faces (30 smiley faces; 20 correct answers on average) and participants are given 20 s per line to tag their choices. Once the 20 s are over, they stop and start with the next line. The number of correctly marked smiley faces is used as the critical attention score.

##### Text reading

Within the text reading task two expository texts with different levels of complexity were presented. The less complex text was about the planet Venus and consisted of a heading and 10 sentences (150 words in total). The second, more scientific text covered the topic of using plants as energy storage. It also had a heading but was only seven sentences long (134 words in total). The German adaptation of the Flesch Reading Ease Scores (Amstad, 1978) for both texts confirmed the differences in readability (easy text: 56, hard text: 43). Additionally, 20 native German students from the FU Berlin (*M*_*age*_ = 22.3, *SD*_*age*_ = 3.9; 13 females) rated both text types on how readable and how interesting they were using five-point scales (1 = low and 5 = high). As assumed, Readability ratings for the text about the planet Venus were significantly higher than Readability ratings for the text about energy storage [*M*_*easytext*_ = 4.8, *SD*_*easytext*_ = 0.6; *M*_*difficulttext*_ = 4.0, *SD*_*difficulttext*_ = 0.7, *t*(19) = 3.92, *p* < 0.001, *d* = 1.16], whereas both text types were rated as equally interesting [*M*_*easytext*_ = 3.9, *SD*_*easytext*_ = 0.9; *M*_*difficulttext*_ = 3.7, *SD*_*difficulttext*_ = 1.2, *t*(19) < 1].

Participants were asked to read the texts silently while their eye movements were recorded. After each text they answered four comprehension questions. The two variables of interest were FFD as a measure of the difficulty of the word’s initial visual recognition as well as lexical access and TRT as a global measure of basic reading ability as well as general comprehension (Hyönä et al., 2003; Boston et al., 2008). Results are reported based at text level analyses, for additional word level analyses see Supplementary Table 3.

#### Sample

The sample consisted of 34 native German speaking participants (24 females) with a mean age of 28.6 years (*SD* = 8.92, range: 19–50) and a mean of 17.3 years (*SD* = 3.43) of formal education, who did not take part in the first study. Participants were recruited within the FU Berlin and outside the FU Berlin via announcements in local newspapers. Prior to testing, participants gave written informed consent. The mean SLS-Berlin score was 59.2 (*SD* = 10.7, range: 39–76), which corresponds to the mean from the age equivalent norm sample, i.e., that of younger adults (see Supplementary Table 3). All participants had normal or corrected-to-normal vision and none had a diagnosed reading or learning disability. They were paid 10 EUR for about 1 h of testing. The study was approved by the ethics committee of the FU Berlin.

#### Procedure

The SLS-Berlin was always administered first in each test session. In contrast to the norm sample, we recorded eye movements during the completion of the SLS-Berlin as well as during silent text reading. For this purpose, participants were seated in front of a 19-inch monitor (120-Hz refresh rate, 1024 × 768 resolution) at a viewing distance of approximately 65 cm. An EyeLink 1000 tower mounted eye tracker build by SR Research (Mississauga, ON, Canada^2^) was used to collect the eye movement data. Sampling frequency was 1,000 Hz. Only the right eye was tracked. Participants’ responses were collected with a standard keyboard, a forehead rest was used to minimize head movements. A nine-point calibration cycle at the beginning of each task (SLS-Berlin, easy text, hard text) and session was used to ensure a spatial resolution error of less than 0.5° of visual angle. All tasks were controlled with Experiment Builder software (RS Research, version 1.10.1630). Font type and size was the same in all tasks. The sentences within the SLS-Berlin and the two expository texts were written in Arial, font size 20, double spaced and left aligned. The procedure for the SLS-Berlin followed the routine described for the norm sample. Due to the font size, long sentences, especially from the second part of the test, were presented on three lines.

After finishing the SLS-Berlin, participants took a rest in which the experimenter described the text reading task. Participants were instructed to silently read each text at their own speed. They were also informed about a short memory test administered directly after reading. Each text presentation started with the calibration described above followed by a fixation cross presented for 500 ms to the left of the first word of the text. Both texts were presented on three pages, the end of a page always coincided with the end of a sentence. Participants proceeded to the next page by pressing the spacebar. At the end of the text reading task, participants were asked four comprehension questions. The easy text was always read first, followed by the more complex text.

The SLRT-II was conducted next with word reading preceding pseudoword reading. For both tasks, participants received a training list of 8 items to be read out loud and corrective feedback if necessary. During the test session, the experimenter noted the number of correctly read words and pseudowords within 1 min.

Finally, at the very end of the entire test session, participants conducted the Smiley-Test. The experimenter explained the task with the help of a short example, followed by a short training session. Then the 3-min test session started. Overall, the whole experimental session, including eye-tracking, took approximately 40 min.

#### Data Analysis

To obtain additional information about the validity of the SLS-Berlin score, we calculated Pearson’s-product-moment correlations between SLS-Berlin scores, the two measures for word and pseudoword identification and the results from the Smiley-Test.

Eye-tracking data was analyzed by first removing participants that had missing data for more than 50% of the words. This led to the exclusion of one subject. For the remaining 33 participants, we then removed fixations shorter than 50 ms or longer than 1000 ms as well as blinks and instances of track-loss. We also excluded data recorded for text headings, initial and final words. The latter was done since words in the beginning and the end of a page and a sentence are often processed differently (Rayner et al., 2000; Kuperman and Van Dyke, 2011). All words with a TRT of zero were handled as missing. This procedure left 4,388 data points for the easy text and 4,038 data points for the more complex text for which each participant’s mean FFD and TRT were finally calculated. Extreme values, i.e., values deviating more than 2.5 standard deviations from an individual’s mean FFD and TRT of each text were removed beforehand (data loss of less than 3.5% for both texts). To assess the predictive power of SLS-Berlin scores for both texts, we conducted linear, quadratic and cubic regression analyses following the same procedure described in Study 1. Due to the small sample size of 33 participants, we did not perform cross-validation.

### Results

#### Comparing the SLS-Berlin With Measures of Word and Pseudoword Recognition as Well as Visual Attention and Processing Speed

On average, the participants read 122.0 words (*SD* = 14.0, range: 84–156) and 80.3 pseudowords (*SD* = 17.9, range: 46–134). Existing norms of the SLRT-II only cover slightly younger adults (high school and university students) and according to these norms, the average word reading score of the present sample corresponds to a percentile rank of 63 (range: 6–99), the average pseudoword reading score to one of 61 (range: 9–99). The Smiley-Test does not have any normative data. Our participants marked on average 249.3 smileys correctly (*SD* = 36.1, range: 175–319). After checking for normal distribution, we calculated Pearson’s product-moment correlations between SLS-Berlin scores and the two measurements for word and pseudoword identification as well as Smiley-Test scores. The highest correlation was observed between SLS-Berlin scores and pseudoword reading: *r*_*SLS–Berlin– SLRT–II pseudoword*_ = 0.59, *t*(32) = 4.18, *p* < 0.001, 95% CI [0.32, 0.78], followed by the correlation with word reading: *r*_*SLS–Berlin– SLRT–II word*_ = 0.51, *t*(32) = 3.38, *p* = 0.002, 95% CI [0.21, 0.73]. The correlation between the SLS-Berlin and the Smiley-Test was weaker and not significant: *r*_*SLS–Berlin –Smiley–Test*_ = 0.23, *t*(32) = 1.32, *p* = 0.18, 95% CI [−0.11, 0.53]. We used the cocor module (Diedenhofen and Musch, 2015) to test whether the correlations between the SLS-Berlin and the two SLRT-II reading tasks were higher than the correlations between the SLS-Berlin and the Smiley-Test. The one-tailed z-test from Hittner et al. (2003) indicated that the correlation coefficient of the SLS-Berlin and pseudoword reading was significantly higher than that of the SLS-Berlin and the Smiley-Test (z_*SLS–Berlin– SLRT–II pseudoword vs. SLS–Berlin –Smiley–Test*_ = 1.99, *p* = 0.02). The comparison of the correlation coefficients of the SLS-Berlin and word reading with the SLS-Berlin and the Smiley-Test failed to reach statistical significance, z_*SLS–Berlin– SLRT–II word vs. SLS–Berlin –Smiley–Test*_ = 1.46, *p* = 0.07.

#### Assessing the Predictive Power of the SLS-Berlin on Eye-Tracking Measures

To investigate the predictive power of the SLS-Berlin with respect to early and late processes related to reading proficiency, we analyzed the error rates for the comprehension questions and calculated FFD and TRT, respectively for both the easy and the more complex text. Mean error rates for the easy text (*M* = 15.4, *SD* = 15.1) were significantly lower [*t*(33) = −6.12, *p* < 0.0001, *d* = 1.15] compared to mean error rates for the difficult text (*M* = 40.4, *SD* = 26.8). Mean FFD (*M*_*easytext*_ = 196.2, *SD_*easytext*_* = 20.4; *M*_*complextext*_ = 204.8, *SD_*complextext*_* = 20.2) as well as mean TRT (*M*_*easytext*_ = 293.4, *SD_*easytext*_* = 54.5; *M*_*complextext*_ = 383.3, *SD_*complextext*_* = 78.1) were significantly longer while reading the difficult text compared to the easy text, *t*_*MeanFFD*_ (32) = 5.56, *p* < 0.001, *d* = 0.40, *t*_*MeanTRT*_ (32) = 8.81, *p* < 0.001, *d* = 1.33. The correlations between mean error rates and SLS-Berlin scores were not significant (both *r*s < −0.18, *t*s(32) < −1.01, *p*s > 0.06; 95% CI_*easy* text_ [−0.56, 0.06], 95% CI_*difficult*_ _*text*_ [−0.48, −0.17]). The following regression analyses (model parameters and summaries are reported in Table 9) indicated decreasing FFD and TRT with increasing SLS-Berlin scores for both text types. With the exception of TRT of the easy text, quadratic models showed best model fits (easy text: *adj. R*^2^_*MeanFFD*_ = 0.24; more complex text: *adj. R*^2^_*MeanFFD*_ = 0.31; *adj. R*^2^_*MeanTRT*_ = 0.24). These quadratic relationships indicated a strong relationship between the eye-tracking measures and the SLS-Berlin score for less skilled readers, i.e., for readers who score below the expected mean norm value of the younger age group. For skilled readers with SLS-Berlin scores above the age equivalent mean norm, the relationship is quite low or absent. For mean TRT of the easy text, we observed a negative linear relationship (*adj. R*^2^*_*MeanTRT*_* = 0.37) indicating a general decrease of TRT with increasing SLS-Berlin scores.

**TABLE 9:** Model parameters and summaries of linear and polynomial regression analyses of SLS-Berlin score predicting eyetracking measures collected for reading an easy and a difficult scientific text.

	**Model parameters**				**Model summary**		
	**Estimate**	***SE***	***t*-value**	***p*-value**	***adj. R*^2^**	***F-*value**	***p*-value**
**Easy text**							
*Mean first fixation duration*							
Linear	–0.74	0.28	–2.56	0.016	−	−	−
Quadratic	0.06	0.04	2.01	0.05	0.24	5.92	0.007
Cubic	−	−	−	−	−	−	−
*Mean total reading time*							
Linear	–3.14	0.7	–4.47	<0.001	0.37	19.97	<0.001
Quadratic	−	−	−	−	−	−	−
Cubic	−	−	−	−	−	−	−
**Difficult text**							
*Mean first fixation duration*							
Linear	–0.85	0.27	–3.11	0.004	−	−	−
Quadratic	0.05	0.03	2.17	0.04	0.31	8.31	0.001
Cubic	−	−	−	−	−	−	−
*Mean total reading time*							
Linear	−2.6^a^	1.10^a^	−2.37^a^	0.03^a^	−	−	−
Quadratic	0.23^a^	0.10^a^	2.01^a^	0.05^a^	0.23^a^	5.77^a^	0.007^a^
Cubic	−	−	−	−	−	−	−

*All models were checked for influential data points and outliers. ^*a*^Best model fit after excluding one participant because of large standardized residual (< ± 2.5).*

### Discussion

In Study 2, we first compared the SLS-Berlin with two measures of basal reading fluency (single word and pseudoword recognition) and one measure of serial allocation of visual attention and discrimination as well as general processing speed. Regarding the multidimensional nature of reading comprehension (Fletcher, 2006), we assumed strong but not perfect positive relationships with the two SLRT-II measures and a significantly weaker correlation with the Smiley-Test. Our results are in line with these assumptions. Number of words and pseudowords correlated significantly with SLS-Berlin scores (*r* = 0.51 and *r* = 0.59). Moreover, both correlations coefficients were descriptively higher than the non-significant correlation between the SLS-Berlin scores and the results of the Smiley-Test. Taken together, these results indicate a mild, if any, influence of general processing speed on SLS-Berlin scores. The absence of a strong correlation between the SLS-Berlin and the Smiley-Test indicates that the problem of the well-documented phenomenon of age-related slowing (Salthouse, 1996, 2000) is not central for the SLS-Berlin. Age-related slowing would imply longer response times and therefore lower SLS-Berlin scores for older compared to younger adults independent of reading proficiency. Yet in line with the non-significant correlation between the SLS-Berlin and the Smiley-Test, observed age effects within the norm sample were relatively small and only significant within the older subsample.

That the SLS-Berlin measures foremost reading proficiency is further confirmed by the text based analyses of the eye-tracking data measured while reading an easy and a difficult text. All four regression analyses using the SLS-Berlin score as a predictor for mean FFD and mean TRT are highly significant and demonstrated that readers with lower SLS-Berlin scores exhibited longer mean FFDs and mean TRTs per word than readers with high SLS-Berlin scores. These results are in line with previous research, i.e., more and longer fixations and TRTs are reported for less skilled and dyslexic readers compared to skilled readers (Ashby et al., 2005; Thaler et al., 2009; Hawelka et al., 2015; Krieber et al., 2016). The additional word level analyses reported in the Supplementary Table 3 also emphasizes the predictive power of the SLS-Berlin. Here, we also observed longer reading times and less skipping for readers with lower SLS-Berlin scores compared to readers with higher scores. In line with Kuperman and Van Dyke (2011), error rates of responses to comprehension questions did not correlate significantly with measures of individual differences in reading proficiency.

The analyses of mean FFD and mean TRT also replicated the well-established effect of significantly longer FFDs and TRTs per word when reading a difficult text as when reading an easy one (Rayner et al., 2006). We therefore investigated separately for each text, how well SLS-Berlin scores relate to FFD and TRT. If the SLS-Berlin, as a sentence-based screening tool, is sensitive to both, basic subprocesses of reading and general comprehension processes necessary for meaning integration and situation model building, the SLS-Berlin score should best predict TRT in the easy text condition. Compared to FFD, which is usually interpreted as being sensitive to early and stronger automatized processes around lexical access (Boston et al., 2008), TRT also entails rereading and backward regressions which are commonly associated with difficulties in linguistic processing and text comprehension processes (Reichle et al., 1998; Engbert et al., 2005). While in the more complex text condition, these later processes may override effects of early and more basic processes (Rayner et al., 2006), TRT in the easy text condition should reflect both early and later processes. The results of our regression analyses using SLS-Berlin scores to predict FFD and TRT in text reading are fully in line with this assumption. We obtained the best model fit (*adj. R*^2^ = 0.37) for the model predicting mean TRT of the easy text. This is also the only regression model for which we observed a strong and positive relationship between SLS-Berlin scores and eye-tracking measures. Correspondingly, the word level analyses of TRT reported in the Supplementary Table 3 show a significant interaction between text type and SLS-Berlin score. The negative relationship between SLS-Berlin and TRT was slightly more pronounced in the easy compared to the difficult text (see Supplementary Figure S3).

**FIGURE 4:**
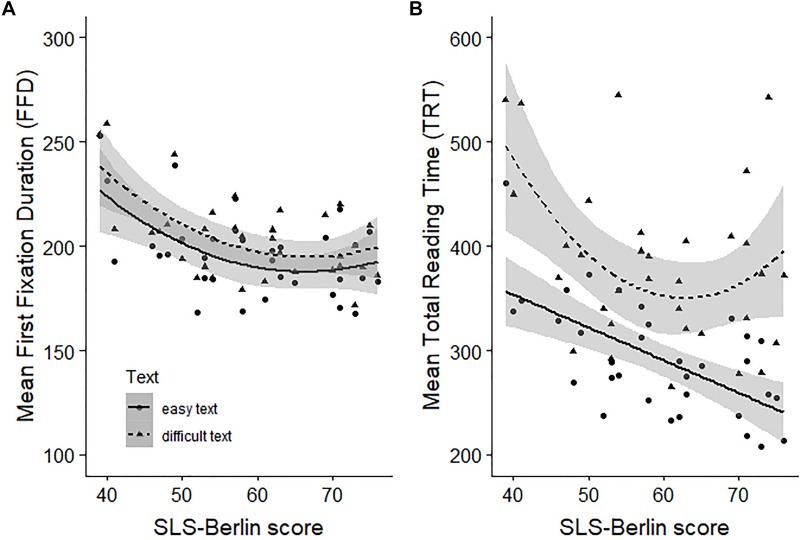
SLS-Berlin Scores predicting Mean First Fixation Duration **(A)** and Mean Total Reading Time **(B)** for reading an easy and a difficult text.

The observed linear relationship in the easy text condition indicates the SLS-Berlin’s sensitivity to differentiate both within the scope of less skilled readers and within the scope of skilled readers. Nevertheless, all other models for which we observed quadratic relationships, suggest that SLS-Berlin scores are more sensitive for distinguishing within less skilled readers as it is within skilled readers. As depicted in Figure 4, for SLS-Berlin scores of 60 and above, the curves flatten. Taken together, comparing SLS-Berlin scores with measures sensitive to early basic processes of reading and measures also sensitive to later comprehension processes underscore the applicability of the SLS-Berlin as a well-suited screening tool to assess individual differences in reading proficiency. The SLS-Berlin seems to be very good in differentiating skilled from less skilled readers whereby its highest sensitivity seems to be within the scope of less skilled readers.

## General Discussion

Within this article, we introduced a computerized screening test for adults to assess individual differences in reading proficiency. The test structure of the SLS-Berlin follows that of a well-established screening test for German children, the SLS (Mayringer and Wimmer, 2003; Auer et al., 2005; Wimmer and Mayringer, 2014) which measures basic reading ability by simultaneously evaluating reading comprehension and reading speed. Even though, a developmental study with German speaking first graders reading word lists suggests reading speed to differentiate fairly well between good and poor readers (Wimmer and Hummer, 1990), word-based measures of reading speed and/or accuracy underrepresent the construct of reading fluency (Cunningham, 2001; Kuhn and Stahl, 2003). As reading proficiency, in our eyes, comprises the successful integration of early and basic processes of word decoding and lexical access as well as higher-order processes of comprehension to establish local and global coherence, a sentence-based screening test, such as the SLS-Berlin should firstly be able to capture both of these essential processes and secondly, differentiate well between and within poor and good adult readers.

### Norms and Validity of the SLS-Berlin

The norm study of the SLS-Berlin attested very high reliability (*r* = 0.95) for the entire range of the norm sample covering the ages of 16 to 88 years. Comparisons of SLS-Berlin scores with other measures of basic reading fluency and eye movement data during text reading supports the notion that the SLS-Berlin is indeed sensitive for both early word decoding and later comprehension processes during reading. The SLS-Berlin is therefore an excellent screening tool to assess reading proficiency across the life span. The screening is just as intended to pave the way for future studies exploring individual differences in reading as it can be used in educational settings. With a duration of around 7 min, the SLS-Berlin fills the gap for a short and easy to use screening tool for reading proficiency, particularly for adult readers of all ages, which is still missing for the German language. The computer-based application allows for easily realizable group testing which can be particularly important in settings that require a large number of participants or when environmental factors limit testing time, for example, in clinical settings. While for these specific instances, norms for patients still need to be established, the SLS-Berlin currently provides information which can be used to distinguish between general reading ability levels. More specifically, if a person yields an SLS-Berlin score well below the expected age specific norm, it may serve as an indication for further investigation.

### Age Related Effects of the SLS-Berlin and Response Times

Although not being the focus of the present article, having collected data of 655 participants below the age of 60 and 1,493 participants being at least 60 years or older, gave us the possibility to further investigate written language processing across the life span in German adults. For this purpose, we analyzed the relationship of SLS-Berlin scores and response times separately for the subsamples of younger and older adults. With the exception of Complexity Ratings and Sentence ID, the order of significant sentence characteristics predicting SLS-Berlin scores by the amount of variance explained was similar for both age groups. However, both Sentence ID and Complexity Ratings explained the most variance in younger and older adults with younger adults relying more heavily on Sentence ID and older adults more on Complexity Ratings. This suggests that both, the younger as well as the older subsample draw in large parts on the same fundamental mechanisms while reading sentences, making the SLS-Berlin an ideal tool for investigating both younger and older readers.

However, there seems to be subtle differences with respect to age when looking at the nature of the relationship between SLS-Berlin scores and response times. As the regression functions differ for five of the seven significant predictors between the younger and older subsample, the nature of the relationship between predictors and sentence reading time seems to slightly change across the life span. Additionally, comparing the *adj. R*^2^ of the two subsamples, the biggest differences in explained variance were found for Number of Words (12% advantage for the older subsample) and Sentence ID (8% advantage for the younger subsample). This yields two important implications. First, in line with previous research regarding single word recognition in both English and German, older adults seem to have no deficits in identifying and assigning meaning to words (e.g., Ratcliff et al., 2004; Spaniol et al., 2006; Froehlich et al., 2016). In fact, it has been reported, that older compared to younger adults spend more time on identifying words (lexical access) to compensate for age related cognitive deficits, such as declines in working memory or processing speed (Stine-Morrow et al., 2008). Second, as the test progresses, the impact of Sentence ID on sentence response time becomes larger for the younger subsample than for the older subsample suggesting possible differences in reading strategies. While younger readers may initially emphasize speed, older readers possibly tend to read the sentences in a more constant speed. With the increasing complexity of the sentences younger adults are forced to read more thoroughly and thus slow down toward the end of the test. Comparing the differences in reading speed (measured in words per minute) and age group between the first and second half of the SLS-Berlin (sentences 1–38 vs. 39–77) seem to confirm this assumption. Reading speed of the younger subsample decelerates to a greater degree than that of the older subsample [younger adults: *M_SLS–Berlin1st –SLS–Berlin2nd_* = 28.2, *SD* = 27.2; older adults: *M_SLS–Berlin1st –SLS–Berlin2nd_* = 11.6, *SD* = 16.1], *t*(2136) = 17.6, *p* < 0.001, *d* = 0.82. However, we acknowledge that this is rather speculative. Given the sparsity of available data for German adults, further research on the mechanics of reading and possible changes in reading strategies across the life span is necessary.

### Limitations and Outlook

The sentences for the SLS-Berlin were constructed to initiate normal reading in an ecologically valid setting while minimizing shallow processing and skim reading. The analyses of the relationships between sentence characteristics and response times of all 77 sentences indicated that this implementation was successful. Nevertheless, the slightly left-skewed distributions of the SLS-Berlin scores suggest better discriminability within poorer than within skilled to very skilled readers. With the intended application in mind, i.e., within reading research, educational and perhaps clinical settings, we are convinced, that the sensitivity of the SLS-Berlin within the lower performance range is adequate. An extension of the test sentences to differentiate more precisely within the upper performance spectrum would further improve the SLS-Berlin. However, prolonging the test would also lead to enhanced test times which would then restrict its applicability as a short and easy to use screening tool.

The chosen time frame of 3 min to calculate the final test score marks the SLS-Berlin as a somewhat speeded screening test. We understand that this is rather critical considering the intended application of the SLS-Berlin across the whole life span. Independently from looking at reading specific processes at word, sentence or text level, factors related to processing speed are among the biological and behavioral variables with the strongest association to age (Salthouse, 1996). Sensorimotor and cognitive slowing increases with increasing age, resulting in generally larger RTs for older adults compared to younger adults (e.g., Salthouse, 1996; Verhaeghen and Salthouse, 1997). To counteract possible disadvantages of especially older participants, the instruction of SLS-Berlin was changed compared to the children’s version. Neither is the 3-min cut-off mentioned nor are the participants interrupted while working on the 77 test sentences. Instead, in the computerized version participants read all sentences within a self-paced paradigm. This allows particularly older readers to use strategies to compensate for processing deficiencies such as greater reliance on discourse context and increased resource allocation (Stine-Morrow et al., 2008). Nevertheless, we estimated the possible influence of processing speed on the SLS-Berlin in Study 2, where we compared SLS-Berlin scores with a measure for general processing speed. The slightly positive yet non-significant correlation (*r* = 0.23, *p* = 0.18) indicated that our adjustments of the instruction were successful. Accordingly, age related differences in SLS-Berlin scores were relatively small and were restricted to the older subsample. Whether the observed decline in SLS-Berlin scores starting around an age of 60 is related to an increase in age related slowing or to changes in reading specific processes cannot be answered within the scope of the present study and should be subject of further research.

In a first step to resolve this issue, Froehlich et al. (2016) investigated the impact of aging on the subprocesses of reading by using hierarchical diffusion modeling. Hierarchical diffusion modeling bears the advantage to disentangle processes underlying decision tasks, such as stimulus encoding (*a priori*) decision making and processing efficiency, i.e., the speed of information uptake (Ratcliff, 1978; Vandekerckhove et al., 2011; Voss et al., 2013). With regard to the latter, results showed that younger adults outperformed older adults (> 60 years) in letter identification and phonological processing. However, with respect to orthographic and semantic processing, younger adults were slower in information uptake than high performing older adults. Yet, low performing older adults performed below the level of younger adults. These results are a firm indication, that besides age, proficiency impacts the degree to which a reader may rely on the different subprocesses of reading. The SLS-Berlin, as it is presented here, treats reading skill as a unitary construct. One important topic for further research is to explore the relationship between single subprocesses of reading and how they are reflected in the SLS-Berlin.

## Conclusion

The very high Spearman-Brown corrected reliability (*r* = 0.95) and the results from Study 2 demonstrate that the SLS-Berlin is a valuable screening tool for the systematic exploration of individual differences in reading proficiency. The test differentiates very well between poor and good readers. The huge number and extensive age range of the norm sample with an emphasis on age groups above 60 ensures the test to be used for a variety of important (developmental) research questions. Further developments are planned to establish a parallel test version as well as increase the relatively small amount of norm data for the age groups between 40 and 60 years of age. However, we think, the SLS-Berlin’s short duration and easy administration makes it a highly potential tool for the investigation of reading abilities in not only underresearched populations such as older adults and individuals with brain lesions but also in developing and proficient readers.

## Data Availability

The raw data supporting the conclusions of this manuscript will be made available by the authors, without undue reservation, to any qualified researcher. The norm data for the SLS-Berlin are part of the Supplementary Table 2. Both, the SLS-Berlin (available for all standard platforms such as Linux, macOS, and Windows as it is written in PsychoPy3; Peirce and MacAskill, 2019) as well as continuously updated norms are available at: https://www.ewi-psy.fu-berlin.de/einrichtungen/arbeitsbereiche/allgpsy/Download/index.html.

## Ethics Statement

This study was approved by the Ethics Commitees of the Max Planck Institute for Human Development, Berlin and the Freie Universität Berlin, Department of Education and Psychology.

## Author Contributions

JL, EF, and AJ contributed to the conception and design of the study. FH developed the test stimuli. JL and EF organized the database, performed the statistical analysis, and wrote the first draft of the manuscript. All authors contributed to the manuscript revision, and read and approved the submitted version.

## Conflict of Interest Statement

The authors declare that the research was conducted in the absence of any commercial or financial relationships that could be construed as a potential conflict of interest.

## Footnotes

1 To assess the relationship between Sentence ID and all sentence characteristics, we used regression analyses as described for the analysis of the response time data in study 1 (see section “Study 1-Data Analysis”). Due to the relative small sample pool of 77 sentences, cross-validation was omitted.

2 http://sr-research.com/pdf/techspec.pdf
