# Supplementary material for: The SLS-Berlin: Validation of a German Computer-Based Screening Test to Measure Reading Proficiency in Early and Late Adulthood
Source: Front Psychol. 2019 Aug 14;10:1682. doi: 10.3389/fpsyg.2019.01682 (PMC6702301; doi:10.3389/fpsyg.2019.01682)
Supplement: Supplementary file 1 [file Table_1.pdf]

Supplementary Material

Test sentences used in the SLS-Berlin

Sentence ID	Sentence	Correct/Incorrect	Mean complexity Rating (1-5)
1	Ein Nashorn ist ein Blechblasinstrument.	incorrect	1
2	Mit einer Waage wird die Körpergröße einer Person gemessen.	incorrect	1.79
3	Wenn man regelmäßig ins Solarium geht, bekommt man eine blasse Hautfarbe.	incorrect	2.16
4	Ein Mobiltelefon ist sehr praktisch, wenn man unterwegs telefonieren will.	correct	1.74
5	Der Mond wurde vor etwa 200 Jahren vom Menschen besiedelt.	incorrect	1.63
6	Aus Sicherheitsgründen ist das Rauchen an Tankstellen verboten.	correct	1.53
7	Gegen Seekrankheit hilft eine Behandlung mit Blumendünger.	incorrect	1.74
8	Betrunkene Autofahrer haben eine verlangsamte Reaktionsgeschwindigkeit.	correct	1.74
9	Gemüse sollte schonend zubereitet werden, damit die Vitamine erhalten bleiben.	correct	2.11
10	Zum Treffen einer Jugendgruppe kommen hauptsächlich Senioren, um Schach zu spielen.	incorrect	2.47
11	Mit einem Mobiltelefon kann man telefonieren und Kurznachrichten versenden.	correct	1.95
12	Bestimmte Medikamente können nur gekauft werden, wenn der Arzt ein Rezept ausstellt.	correct	1.79
13	In fünf Sterne Luxushotels muss man seinen eigenen Schlafsack mitnehmen.	incorrect	2.26
14	Damit man berechtigt ist, ein Auto zu fahren, benötigt man einen Führerschein.	correct	2
15	Einen Hamburger isst man, wenn man sich besonders gesund und bewusst ernähren möchte.	incorrect	2.21
16	Eine Röntgenuntersuchung ermöglicht es, einen Knochenbruch festzustellen.	correct	1.95
17	Cornflakes sind knusprige Flocken aus zerkleinertem, geröstetem Straßensplitt.	incorrect	2.11
18	Eine Sommergrippe ist eine Krankheit, die durch Radio hören übertragen werden kann.	incorrect	2.26
19	Kinder, die eine außergewöhnliche Begabung haben, nennt man Wunderkinder.	correct	2.21
20	Die Straßenverkehrsordnung wurde speziell für die Luftfahrt geschaffen.	incorrect	1.75

21	Tennisspieler benötigen, um ihren Sport auszuüben, einen Swimmingpool oder einen Badesee.	incorrect	2.84
22	Um schmutzige Wäsche wieder sauber zu bekommen, gibt man sie in den Geschirrspüler.	incorrect	2.21
23	Bei Versandhäusern kann man die gewünschten Waren auch telefonisch bestellen.	correct	1.89
24	Eine Gehirnerschütterung kann man bekommen, wenn man hart mit dem Kopf aufschlägt.	correct	2.16
25	Unter Subtrahieren versteht man das Zusammenzählen von verschiedenen Zahlen.	incorrect	2
26	Wenn man an Grippe erkrankt ist, sollte man am besten im Bett bleiben und sich auskurieren.	correct	2.32
27	Mischt man die Farbe Blau und die Farbe Gelb zusammen, erhält man Grün.	correct	2.11
28	Schädlingsbekämpfungsmittel können Gesundheitsschäden auslösen.	correct	1.89
29	Um als Arzt erfolgreich arbeiten zu können, ist eine hohe musikalische Begabung erforderlich.	incorrect	2.42
30	Feinschmecker essen gerne köstliche Lebensmittel und trinken oft guten Wein zu ihrem Essen.	correct	2.47
31	Der Zentimeter ist eine sehr gebräuchliche Einheit zur Messung des Gewichts.	incorrect	1.74
32	Beim Auftreten von Vergiftungserscheinungen ist es ratsam, einen Arzt zu kontaktieren.	correct	1.79
33	In einem Labyrinth findet man gute Ausschilderungen, die zum Ziel führen.	incorrect	2.42
34	Das Fernsehen ist ein Massenmedium, das in praktisch jedem Haushalt zu finden ist.	correct	2.16
35	Ein Hauptaufgabengebiet der Feuerwehr ist es, Brände zu legen.	incorrect	1.63
36	Intoleranz legt jemand an den Tag, wenn er ein begabter Handwerker ist.	incorrect	2.37
37	Für Arbeiten in der Hochseefischerei trägt man am besten elegante Herrenkleidung.	incorrect	2
38	Für häufig benutzte Redewendungen ist unbedingt eine Haftpflichtversicherung abzuschließen.	incorrect	2.47
39	Bei einer Bewerbung kann unter anderem ein Empfehlungsschreiben hilfreich sein.	correct	2
40	Programme, die heimlich in Computer eindringen und diesen schachmatt setzen können, nennt man Viren.	correct	3.21
41	Eine Straßenbahnhaltestelle ist ein beliebter Zwischenstopp für Kleinflugzeuge.	incorrect	2
42	In einem Krankenhaus sind rund um die Uhr viele Krankenpflegerinnen und Ärzte beschäftigt.	correct	2.16
43	Gardinen hängt man vor das Fenster, um andere daran zu hindern, Mittagessen zu kochen.	incorrect	2.53
44	In jeden Verbandskasten gehören ein Fieberthermometer und eine Reiseschreibmaschine.	incorrect	2.63

45	Sonne und angenehme Temperaturen zeichnen eine Schlechtwetterperiode aus.	incorrect	2.05
46	Die Aufgabe der Weltgesundheitsorganisation ist die Ausrichtung von Schönheitswettbewerben.	incorrect	1.84
47	Bei der Herstellung der elektronischen Bauteile von Computern ist höchste Präzision notwendig.	correct	2.26
48	Um an einer Hochschule studieren zu können, benötigt man als Voraussetzung unbedingt eine Schreinerlehre.	incorrect	2.79
49	Ein Lokomotivführer eines Hochgeschwindigkeitszuges sollte unbedingt ein Training im Schuhputzen absolvieren.	correct	2.53
50	Eine Anwendungsmöglichkeit von Fernbedienungen ist das Entzünden von Kerzen.	incorrect	2.05
51	„Warenimport“ bedeutet, dass Güter aus dem Ausland eingeführt werden.	correct	2.32
52	Eine Oper beginnt normalerweise mit dem Spiel der Ouvertüre durch das Küchenteam.	incorrect	2.26
53	Wenn man das Körpergewicht verringern will, dann sollte man die Kalorienzufuhr drosseln.	correct	2.28
54	In lang anhaltenden Trockenperioden ist die Feuerwehr in erhöhter Alarmbereitschaft.	correct	2.21
55	Die Entstehungsgeschichte unserer Erde wird im Branchenverzeichnis anschaulich dargestellt.	incorrect	2.05
56	An den Hauptbahnhöfen großer Städte befinden sich oft Gepäckaufbewahrungen.	correct	1.63
57	Flüchtigkeitsfehler lassen sich durch aufmerksame Arbeit vermeiden.	correct	1.95
58	Eine motorbetriebene Maschine, die Tragflächen hat und fliegen kann, nennt man Kaffeemaschine.	incorrect	2.53
59	Benutzerfreundliche Gebrauchsanleitungen erleichtern die Inbetriebnahme von technischen Geräten.	correct	2.37
60	Bohrinseln, die Öl zu tage fördern, befinden sich ausschließlich im Hochgebirge.	incorrect	2.37
61	Ein beliebter Gesprächsgegenstand auf Generalversammlungen sind Indianergeschichten.	incorrect	1.89
62	Strafverteidiger sind bei ihren Auftritten vor Gericht auf eine gute Gesangausbildung angewiesen.	incorrect	2.74
63	Aneinanderhängende Waggons, die von einer Lokomotive auf Eisenbahnschienen gezogen werden, nennt man Kinderwägen.	incorrect	2.68
64	Die Kundenzufriedenheit hat in modernen Dienstleistungsunternehmen einen hohen Stellenwert.	correct	2.26
65	In heißen Sommernächten können einem Moskitos mit ihrem Surren und ihrem Stechen den Schlaf rauben.	correct	3.16
66	Um Umweltverschmutzung zu vermeiden, ist es sinnvoll den Müll zu trennen und öffentliche Verkehrsmittel zu benutzen.	correct	3.16
67	Die einflussreichsten Informationsquellen für Zeitungsredaktionen sind oftmals Garderobenständer.	incorrect	2.21
68	Der Strom aus dem Elektrizitätswerk wird unter anderem über Hochspannungsleitungen geliefert.	correct	2.16

69	Alle mystischen Phänomene, die man optisch und akustisch wahrnehmen kann, werden üblicherweise in einem Reiseführer zusammengefasst.	incorrect	3.68
70	Abenteuerlustige Menschen fahren gerne in weit entfernte und unbekannte Länder, um dort ihren Urlaub zu verbringen.	correct	2.79
71	Von Versicherungsgesellschaften kann man Informationsmaterial über Hausratsversicherungen erhalten.	correct	2.53
72	Konzentrationsfähigkeit ist etwas, das man vor allen Dingen braucht, um der faszinierenden Tätigkeit des Schlafens nach zu gehen.	incorrect	3.58
73	Vor jeder Operation findet ein Aufklärungsgespräch statt, in dem der Anästhesist über die Risiken von Ampelkreuzungen aufklärt.	incorrect	3.26
74	In unwegsamen Gelände schützen feste Schuhe aus dickem Leder vor Verletzungen, indem sie das Sprunggelenk stützen.	correct	3.47
75	Die schöne Geräuschkulisse von Hufgetrappel von Wildpferden kann man besonders häufig in Großstadtvierteln hören.	incorrect	3.10
76	Bei einem Symposium folgt dem Vortrag eines Referenten über kontroverse Inhalte gewöhnlich ein Wettrennen.	incorrect	3.05
77	Bei sportlichen Aktivitäten empfehlen sich Kleidungsstücke aus funktionellen Materialien, die schnell trocknen und besonders reißfest sind.	correct	3
