# Supplementary material for: The SLS-Berlin: Validation of a German Computer-Based Screening Test to Measure Reading Proficiency in Early and Late Adulthood
Source: Front Psychol. 2019 Aug 14;10:1682. doi: 10.3389/fpsyg.2019.01682 (PMC6702301; doi:10.3389/fpsyg.2019.01682)
Supplement: Supplementary file 2 [file Table_2.pdf]

Supplementary Material

Norm data for the SLS-Berlin

Percentile ranks indicating the percentage of persons from the comparison group with the same or a lower SLS-Berlin score for the whole norm sample, the two subsamples of younger and older adults and for age decades within the subsample of older adults

		All	Subgroups		Age Decades within the Subgroup of Older Adults		
			Younger Adults	Older Adults	60er	70er	80er
		(N=2148)	(N=655)	(N=1493)	(N=663)	(N=832)	(N=25)
**SLS-Berlin Score**	**Age range**	**16-88**	**16-59**	**60-88**	**60-69**	**70-79**	**80-88**
19		0.05	-	0.07	0.16	-	-
20		-	-	-	-	-	-
21		0.14	-	0.20	-	0.12	4
22		0.19	-	0.27	-	0.24	-
23		0.23	-	0.33	-	0.36	-
24		-	-	-	-	-	-
25		-	-	-	-	-	-
26		0.42	-	0.60	-	0.84	-
27		0.51	-	0.74	0.16	1.08	-
28		0.65	0.15	0.87	0.31	1.20	-
29		0.98	0.31	1.27	0.79	1.56	-
30		1.16	-	1.54	1.10	1.68	8
31		1.49	0.46	1.94	1.26	2.16	12
32		2.00	1.07	2.41	1.89	2.52	12
33		2.28	-	2.81	2.20	3.00	12
34		2.75	1.22	3.42	2.36	3.97	12
35		3.26	-	4.15	2.83	4.93	12
36		4.24	1.83	5.29	3.30	6.49	16
37		5.07	2.14	6.36	3.77	7.81	24
38		6.66	3.05	8.24	5.35	9.98	24
39		8.10	4.43	9.71	6.45	11.78	24
40		9.40	4.58	11.52	7.86	13.94	24
41		11.22	5.80	13.60	9.12	16.71	24
42		13.50	6.41	16.61	11.48	20.31	24
43		15.32	7.18	18.89	13.99	22.48	24
44		17.50	9.16	21.17	16.35	24.76	24

45	20.11	11.30	23.98	18.87	27.88	24
46	22.67	13.13	26.86	21.38	31.01	28
47	25.47	15.27	29.94	23.58	34.86	28
48	28.21	17.10	33.09	26.57	38.22	28
49	31.19	18.93	36.57	29.87	41.83	32
50	33.94	21.53	39.38	32.39	44.95	32
51	36.87	24.12	42.46	35.53	48.08	32
52	40.08	27.33	45.68	38.52	51.44	36
53	42.88	31.15	48.02	41.35	53.49	36
54	46.18	34.50	51.31	44.34	56.73	48
55	49.30	36.79	54.79	48.27	59.86	52
56	52.19	39.54	57.74	51.26	62.62	60
57	55.26	42.44	60.88	53.46	66.34	68
58	58.61	45.95	64.17	57.86	68.87	68
59	61.82	48.40	67.72	61.64	72.36	68
60	64.25	50.99	70.06	63.84	74.88	68
61	66.95	53.89	72.67	66.67	77.28	72
62	70.34	57.56	75.95	70.28	80.41	72
63	73.04	59.85	78.83	73.27	83.05	80
64	76.07	62.44	82.05	76.89	86.06	80
65	78.40	64.89	84.33	80.03	87.62	84
66	80.63	67.48	86.40	82.08	89.78	84
67	83.47	70.99	88.95	85.69	91.59	84
68	85.01	73.44	90.09	86.79	92.55	92
69	86.87	76.34	91.49	88.21	93.99	92
70	89.11	79.39	93.37	90.88	95.31	92
71	91.20	82.90	94.84	93.24	96.15	92
72	92.50	85.04	95.78	94.34	96.99	92
73	93.99	87.79	96.72	95.28	97.84	96
74	95.58	90.53	97.79	96.70	98.56	100
75	97.21	93.89	98.66	97.96	99.16	-
76	98.65	96.79	99.46	98.74	100.00	-
77	100.00	100.00	100.00	100.00	-	-
