# Supplementary material for: The SLS-Berlin: Validation of a German Computer-Based Screening Test to Measure Reading Proficiency in Early and Late Adulthood
Source: Front Psychol. 2019 Aug 14;10:1682. doi: 10.3389/fpsyg.2019.01682 (PMC6702301; doi:10.3389/fpsyg.2019.01682)
Supplement: Supplementary file 3 [file Table_3.pdf]

Supplementary Material

1 Additional analyses of the eyetracking data from Study 2

The analyses of the eyetracking data reported in Study 2 demonstrated significant differences for mean first fixation duration (FFD) and mean total reading times (TRT) between an easy and a difficult text. Shorter mean FFD and mean TRT were observed for the easy text compared to the difficult text. Moreover, text level analyses also demonstrated the predictive power of the SLS-Berlin for both text versions. Shorter mean FFD and mean TRT were observed for participants with higher SLS-Berlin scores compared to participants with lower SLS-Berlin scores. Here, we report additional analyses at the level of single words, testing whether SLS-Berlin scores and the type of text were suited to predict the two duration based measures FFD and TRT and skipping. Analyses at the single word level also allow for testing interactions between the SLS-Berlin score and lexical features of words such as word frequency.

1.1 Data analyses

Statistical analyses for FFD and TRT at single word level were conducted using linear mixed effects regression models. These were run in R version 3.5.1 (R Core team, 2014) using the lme4 package (Bates et al., 2015). Values for the durational eye-movement measures FFD and TRT were logarithmized. Before calculating the mixed models for FFD and TRT, all values with more than 2.5 standard deviations above or below the individual mean per participant and text were eliminated as extreme values (2.33-2.56 % of the data). Word skipping, coded binary as ‘yes’ or ‘no’, was analyzed using a logistic linking function (Jaeger, 2008). For the sake of conciseness, only significant tests associated with fixed effects are reported. Fixed effects were analyzed with the Anova function in the car package and Type III sum of squares statistic.

1.1.1 Fixed effects

To examine the effect of SLS-Berlin scores and text type at the single word level, we used both variables as fixed effects as well as word frequency and word type. The rationale for adding the latter two was as follows. First, eye tracking studies about reading literary and non-literary texts demonstrated that word length and word frequency are two of the most important predictors for TRT, number of fixations and skipping (e.g., Just and Carpenter, 1980; Inhoff and Rayner, 1986; Raney and Rayner, 1995; Rayner et al., 1996, Xue et al., 2019). However, both variables are highly correlated increasing the risk of multicollinarity (e.g. Kliegl et al., 1982). In both texts used in Study 2, the correlation between logarithmized word frequency and word length was .79 or .82, depending on the way the logarithmized word frequency was calculated. Two avoid multicollinarity, we therefore included only logarithmized word frequency as fixed effect. We calculated the logarithmized word frequencies for word-forms so that words with different inflections were handled as different cases (the easy text includes 106 different word forms, the difficult text 102). Furthermore, we included only word-forms for which logarithmized word frequencies were available based on the German SUBTLEX-DE (Brysbaert et al., 2011), resulting in 171 word-forms for both texts. To ensure that frequency effects were not mere results of the chosen frequency measure, we recalculated all mixed models using lemma based logarithmic word frequencies according to dlexDB (Heister et al., 2011). These additional calculations produced similar results. Second, empirical research about skipping demonstrated that short words with high frequency and high predictability

are skipped more often than low frequent words with more letters and lower predictability (Angele and Rayner, 2013; Drieghe et al., 2004; Rayner, 1998; 2009). Since short, frequent and high predictive words are function words whereas longer, less frequent and less predicted words are content words, word type was included. The final models for the analyses at single word level included SLS-Berlin score, text type, logarithmized word frequency and word type together with the interaction between the later three variables and the SLS-Berlin score.

To avoid collinearity and to maximize likelihood of model convergence, continuous variables were centered prior to analysis (Baayen, 2008). For the two nominal variables text type and word type effect coding was used.

1.1.2 Random effects

All models include subjects and word-forms nested in text as random effects.

1.2 Results

1.2.1 First Fixation Duration (FFD)

1.2.1.1 Model

m.ffd = lmer (log(FFD) ~ 1 + SLS-Berlin score + logf + text type + word type + SLS-Berlin score * logf + SLS-Berlin score * text type + SLS-Berlin score * word type + (1|subject) + (1|text:word-forms), data, REML=TRUE)

1.2.1.2 Results and Discussion

As reported in **Table S1** and corresponding to the results at text level, only the main effect for SLS-Berlin score and one the interaction between SLS-Berlin score and logarithmized word frequency with the SLS-Berlin score were significant corresponding to the results at text level. The SLS-Berlin predicted significantly FFD at the level of single words.

TABLE S1 | Model parameters and summaries of linear mixed model predicting logarithmized First fixation duration (FFD) at single word level

Predictor	*Chi square*	*Df*	*P*
Intercept	82869.65	1	<.0001****
SLS-Berlin score[a]	7.91	1	.004**
log. word frequency (logf)[a,c,d]	0.02	1	.87
text type[b]	0.34	1	.56
word type[b]	0.27	1	.60
SLS-Berlin score[a] * logf	8.67	1	.003**
SLS-Berlin score[a] * text type[b]	0.45	1	.50
SLS-Berlin score[a] * word type[b]	1.42	1	.23

The model fit for the above mentioned mixed model is: AIC = 4149.85 (for the model including lemma based word frequency AIC = 4806.19). [a] Continuous predictor variables were centred to enhance the interpretability of the mixed model. [b] Categorical predictor variables were included using effect coding. [c] Different inflections of one lemma were handled as unique word-forms. [d] Logarithmic word frequencies for unique word-forms were calculated using the German SUBTLEX-DE (Brysbaert et al., 2011).

As visible in **Figure S1**, participants scoring low on the SLS-Berlin had longer FFD compared to participants scoring high on the SLS-Berlin. Moreover, participants scoring high on the SLS-Berlin showed a slightly negative relationship with word frequency. These participants had slightly shorter FFD for high frequent words compared to low frequent words.

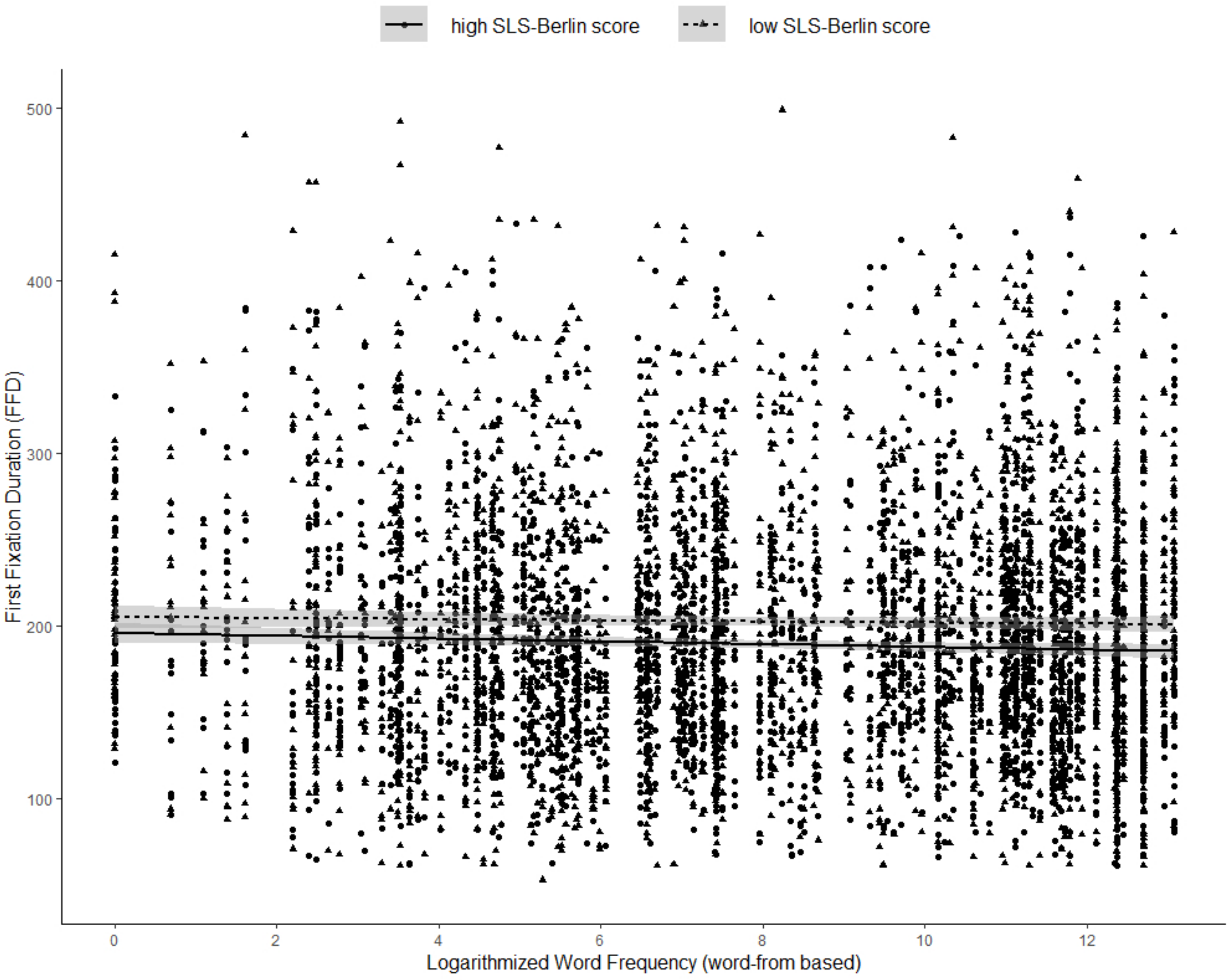

FIGURE S1 | Logarithmized word frequency values predicting First fixation duration (FDD) at single word level grouped by participants with low SLS-Berlin scores (below 59) and high SLS-Berlin scores (above 60). Each data point represents the FFD of a single word and participant.

1.2.2 Total Reading Time (TRT)

1.2.2.1 Model:

m.trt = lmer (log(TRT) ~ 1 + SLS-Berlin score + logf + text type + word type + SLS-Berlin score * logf + SLS-Berlin score * text type + SLS-Berlin score * word type + (1|subject) + (1|text:word-forms), data, REML= TRUE)

1.2.2.2 Results and Discussion

As depicted in **Table S2**, for all predictors, except for word type, significant main effects were observed. Moreover, we also observed a significant interaction between SLS-Berlin scores and text type. As visible in **Figure S2**, participants had shorter TRT for words with higher frequencies compared to words with lower frequencies in both text types which corresponds to the often reported effect of word frequency (e.g., Just and Carpenter, 1980; Inhoff and Rayner, 1986; Raney and Rayner, 1995; Rayner et al., 1996; Pynte et al., 2008; Xue et al., 2019). In accordance with the text level analyses reported in Study 2, both the SLS-Berlin scores as well as text type had an impact on the TRT of single words. Participants scoring low on the SLS-Berlin showed longer TRT for words compared to participants scoring high on SLS-Berlin. Figure S2 depicts the interaction of SLS-Berlin scores separately for the easy and the difficult text. The negative relationship between TRT and SLS-Berlin score is slightly more pronounced in the easy compared to the difficult text.

TABLE S2 | Model parameters and summaries of linear mixed model predicting logarithmized Total Reading Time (TRT) on single word level

Predictor	*Chi square*	*Df*	*p*
Intercept	33371.53		<.0001****
SLS-Berlin score[a]	13.00	1	.0003***
log. word frequency (logf)[a,c,d]	71.58	1	<.0001****
text type[b]	8.97	1	.002**
word type[b]	0.01	1	.91
SLS-Berlin score[a] * logf	0.34	1	.56
SLS-Berlin score[a] * text type[b]	5.32	1	.02*
SLS-Berlin score[a] * word type[b]	0.30	1	.59

The model fit for the above mentioned mixed model was AIC = 8470.47 (for the model including lemma based word frequency AIC = 9795.89). [a] Continuous predictor variables were centred to enhance the interpretability of the mixed model. [b] Categorical predictor variables were included using effect coding. [c] Different inflections of one lemma were handled as unique word-forms. [d] Logarithmic word frequencies for unique word-forms were calculated using the German SUBTLEX-DE (Brysbaert et al., 2011).

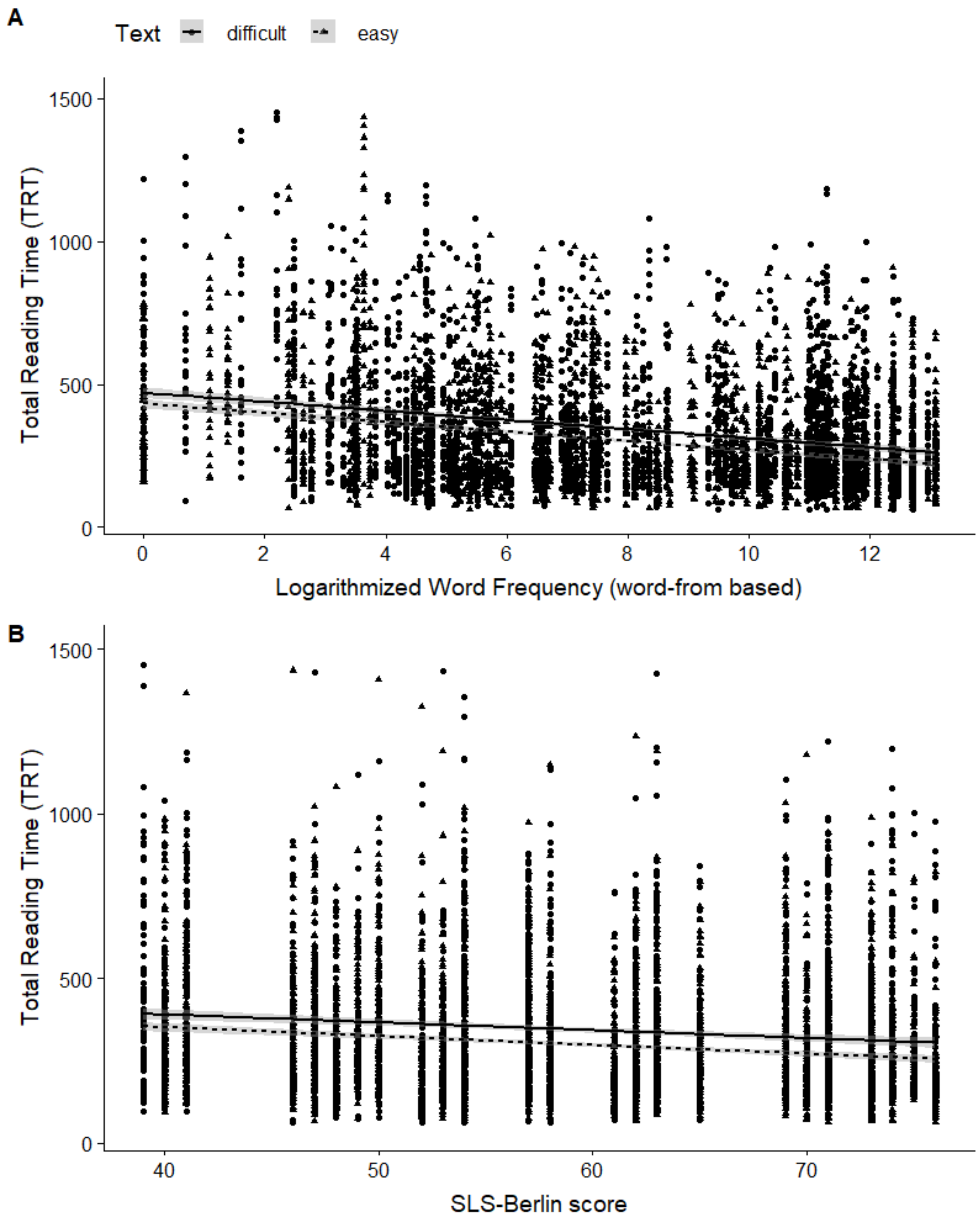

FIGURE S2 | Logarithmized word frequency values predicting Total Reading Time (TRT) at single word level for the easy and the difficult text (**A**) and SLS-Berlin scores predicting TRT at single word level for the easy and the difficult text (**B**). Each data point represents the TRT of a single word and participant.

1.2.3 Skipping

1.2.3.1 Model

m.skip = glmer (Skipping ~ 1 + SLS-Berlin score + logf + text type + word type + SLS-Berlin score * logf + SLS-Berlin score * text type + SLS-Berlin score * word type + (1|subject) + (1|text:word-forms), data, family=binomial("logit"))

1.2.3.2 Results and Discussion

In line with the analyses of the durational eye-movement measures FFD and TRT at text and single word level, the SLS-Berlin significantly predicted word skipping (see **Table S3**). As depicted in **Figure S3,** participants scoring higher on SLS-Berlin skipped words more often than participants scoring lower on SLS-Berlin, an effect also reported by Eskenazi and Folk (2015). Similarly, the analysis of TRT replicated the well-known word frequency effect. High frequent words were skipped more often than low frequent words. No significant interactions with the SLS-Berlin were observed.

TABLE S3 | Model parameters and summaries of linear mixed model predicting skipping binary coded as ‘yes’ or ‘no’

Predictor	*Chi square*	*Df*	*p*
Intercept	266.33	1	<.0001****
SLS-Berlin score[a]	12.64	1	.001 ***
log. word frequency (logf)[a,c,d]	78.61	1	<.0001****
text type[b]	0.21	1	.65
word type[b]	0.94	1	.33
SLS-Berlin score[a] * logf	0.71	1	.40
SLS-Berlin score[a] * text type[b]	0.59	1	.44
SLS-Berlin score[a] * word type[b]	0.03	1	.87

The model fit for the above mentioned mixed model was: AIC = 6532.43 (for the model including lemma based word frequency AIC = 7338.86). [a] *Continuous predictor variables were centred to enhance the interpretability of the mixed model.* [b] *Categorical predictor variables were included using effect coding.* [c] *Different inflections of one lemma were handled as unique word-forms.* [d] *Logarithmic word frequencies for unique word-forms were calculated using the German SUBTLEX-DE (Brysbaert et al., 2011).*

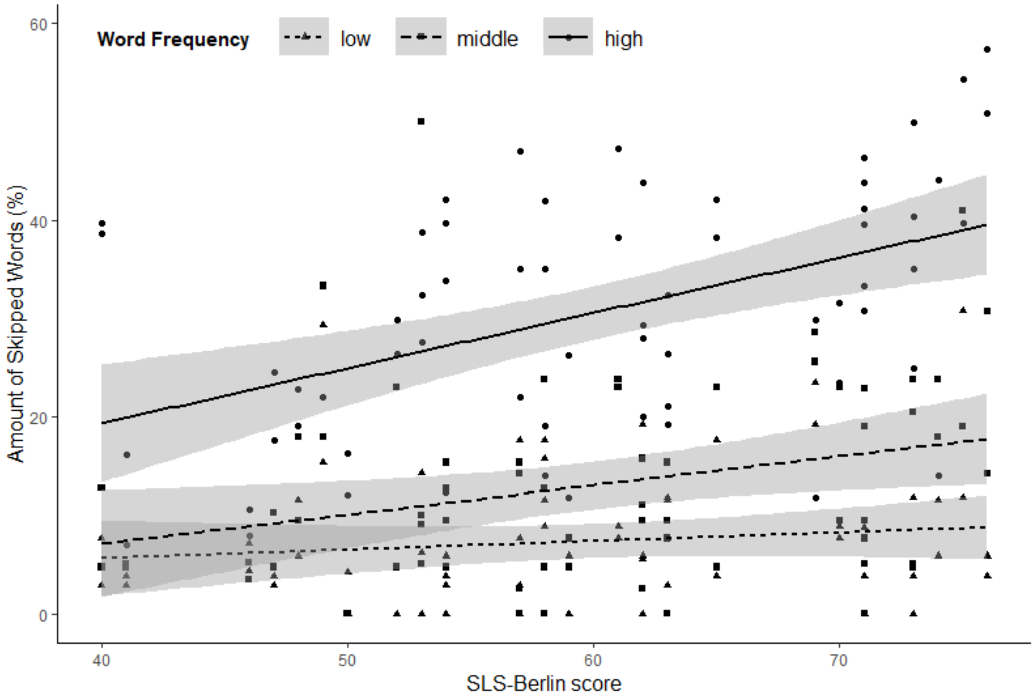

FIGURE S3 | SLS-Berlin scores predicting the amount of skipping (in percent) for low middle and high frequent words (word-form based frequencies). Each data point represents the amount of skipping for one word-frequency-group per text and participant.

1.3 General discussion

The analyses at single word level are in line with the results at text level reported in Study 2. For both durational eye-movement measures and for skipping, a significant effect of the SLS-Berlin was observed emphasizing the aptness of the SLS-Berlin to assess general reading ability. The fact that only the SLS-Berlin but not well established lexical features such as word frequency were significant in all three analyses at single word level are in line with the results of Kuperman and Van Dyke (2011). The researchers were the first demonstrating that differences in reading proficiency account for more variance in readers' eye movement behaviors than word level variables such as frequency and word length. In contrast to Kuperman and Van Dyke, we observed no substantial interaction between word frequency and reading skill, neither for TRT nor for skipping. Only for FFD, a small but significant effect was observed. However, this effect is contrary to other empirical results reporting stronger frequency effects for less skilled readers in eyetracking measures (e.g., Ashby et al., 2005; Hawelka et al., 2010). We observed no evidence for the assumed reduced sensitivity to word frequency for readers with exceptional reading skills (e.g. Chateau and Jared, 2000). Whether the absence of substantial word frequency-by-reading ability interaction is specific for the German language or whether it relies on the corpus style method used to examine fixation times on single words within a passage needs to be tested in upcoming studies. In conclusion, the SLS-Berlin offers the possibility to conduct further eyetracking studies about individual differences in adult German readers.

2 References

Angele, B., and Rayner, K. (2013). Processing the in the parafovea: Are articles skipped automatically? *J. Exp. Psychol. Learn. Mem. Cogn.* 39, 649–662. doi: 10.1037/a0029294.

Ashby, J., Rayner, K., and Clifton, C. (2005). Eye movements of highly skilled and average readers: Differential effects of frequency and predictability. *Q. J. Exp. Psychol. Section A* 58, 1065–1086. doi: 10.1080/02724980443000476.

Baayen, R. H. (2008). *Analyzing Linguistics Data: A Practical Introduction to Statistics*. Cambridge: Cambridge University Press. doi: 10.1017/CBO9780511801686

Bates, D., Maechler, M., Bolker, B., and Walker, S. (2015). Fitting linear mixedeffects models using lme4. J. Statist. Softw. 67, 1–48. doi: 10.18637/jss.v067.i01

Brysbaert, M., Buchmeier, M., Conrad, M., Jacobs, A.M., Bölte, J., and Böhl, A. (2011). The word frequency effect: A review of recent developments and implications for the choice of frequency estimates in German. *Exp. Psychol.* 58, 412-424. doi:10.3758/BRM.41.4.977

Chateau, D. and Jared, D. (2000). Exposure to print and word recognition processes. *Mem. Cogn.* 28(1), 43–153. doi: 10.3758/BF03211582

Drieghe, D., Brysbaert, M., Desmet, T., and De Baecke, C. (2004). Word skipping in reading: On the interplay of linguistic and visual factors. *Eur. J. Cogn. Psychol.* 16, 79–103. doi: 10.1080/09541440340000141

Hawelka, S., Gagl, B., and Wimmer, H. (2010). A dual-route perspective on eye movements of dyslexic readers. *Cogn.* 115(3), 367–379. doi: 10.1016/j.cognition.2009.11.004

Heister, J., Würzner, K.-M., Bubenzer, J., Pohl, E., Hanneforth, T., Geyken, A., and Kliegl, R. (2011). dlexDB - eine lexikalische Datenbank für die psychologische und linguistische Forschung. *Psychol. Rundschau* 62(1), 10-20. doi: 10.1026/0033-3042/a000029

Inhoff, A. W., and Rayner, K. (1986). Parafoveal word processing during eye fixations in reading: Effects of word frequency. *Percept. Psychophysics* 40(6), 431–439. doi: 10.3758/BF03208203

Jaeger, T. F. (2008). Categorical data analysis: away from ANOVAs (transformation or not) and towards Logit Mixed Models. *J. Mem. Lang.* 59, 434–446. doi: 10.1016/j.jml.2007.11.007

Just, M. A., and Carpenter, P. A. (1980). A theory of reading: From eye fixations to comprehension. *Psychol. Rev.* 87(4), 329. doi: 10.1037/0033-295X.87.4.329

Kliegl, R., Olson, R. K., and Davidson, B. J. (1982). Regression analyses as a tool for studying reading processes: Comment on Just and Carpenters eye fixation theory. *Mem. Cogn.* 10(3), 287–296. doi: 10.3758/BF03197640

Kuperman, V., and Van Dyke, J. A. (2011). Effects of individual differences in verbal skills on eye-movement patterns during sentence reading. *J. Mem. Lang.* 65(1), 42–73. doi: 10.1016/j.jml.2011.03.002

R Core team. (2014). *R: A Language and Environment for Statistical Computing*. Vienna. Available online at: http://www.r-project.org

Raney, G. E., and Rayner, K. (1995). Word frequency effects and eye movements during two readings of a text. *Can. J. Exp. Psychol.* 49(2), 151. doi: 10.1037/1196-1961.49.2.151

Rayner, K. (1998). Eye movements in reading and information processing: 20 years of research. *Psychol. Bull.* 124, 372–422. doi: 10.1037/0033-2909.124.3.372

Rayner, K. (2009). The thirty-fifth Sir Frederick Bartlett lecture: Eye movements and attention in reading, scene perception, and visual search. *Q. J. Exp. Psychol.* 68, 1457–1506. doi: 10.1080/17470210902816461

Rayner, K., Sereno, S. C., and Raney, G. E. (1996). Eye movement control in reading: A comparison of two types of models. *J. Exp. Psychol. Hum. Perc. Perform.* 22, 1188–1200. doi: 10.1037/0096-1523.22.5.1188

Xue, S., Lüdtke, J., Sylvester, T., and Jacobs, A. M. (2019). Eye Tracking Shakespeare Sonnets: Combining Quantitative Narrative Analysis and Predictive Modeling. *J. Eye Mov. Res.*, doi: 10.16910/jemr.12.5.2
